# Hepatic arterial infusion chemotherapy versus systemic therapy for advanced hepatocellular carcinoma: a systematic review and meta-analysis

**DOI:** 10.3389/fonc.2023.1265240

**Published:** 2023-10-10

**Authors:** Hyeon-Jong Kim, Seung Hyuk Lee, Hyun Jeong Shim, Hyun Jin Bang, Sang Hee Cho, Ik-Joo Chung, Eu Chang Hwang, Jun Eul Hwang, Woo Kyun Bae

**Affiliations:** ^1^ Division of Hematology-Oncology, Department of Internal Medicine, Chonnam National University Medical School and Hwasun Hospital, Hwasun, Republic of Korea; ^2^ Department of Urology, Chonnam National University Medical School, Hwasun, Republic of Korea

**Keywords:** intra-arterial infusions, carcinoma, hepatocellular carcinoma, drug therapy, survival, GRADE approach (MeSH)

## Abstract

**Introduction:**

To investigate the effects of hepatic arterial infusion chemotherapy (HAIC) with or without systemic chemotherapy compared to systemic chemotherapy alone in patients with locally advanced hepatocellular carcinoma (HCC).

**Methods:**

Following a registered protocol (PROSPERO 2023 CRD42023386780 Available from: https://www.crd.york.ac.uk/prospero/display_record.php?ID=CRD42023386780), a comprehensive search was performed using reputable databases and registries up to December 26, 2022, with no language, publication date, or status restrictions. Only randomized controlled trials (RCTs) investigating the effects of HAIC with or without systemic chemotherapy versus systemic therapy alone were included. The primary outcomes were overall survival (OS), progression-free survival (PFS), and adverse events. The secondary outcomes included the objective response rate (ORR) and disease control rate (DCR). A random-effects model was used, and the certainty of the evidence was rated using GRADE.

**Results:**

Seven RCTs involving 1,010 patients were included. All trials utilized sorafenib as the comparator. Five trials (690 patients) compared HAIC plus sorafenib to sorafenib alone, while two trials (320 patients) compared HAIC to sorafenib. The results indicate that HAIC, with or without sorafenib, may increase OS, PFS, and ORR compared with sorafenib alone. HAIC may enhance DCR, but the evidence is very uncertain. Adverse events were comparable between HAIC plus sorafenib and sorafenib alone. However, adverse events might be decreased in HAIC alone.

**Discussion:**

HAIC with or without systemic chemotherapy may improve survival outcomes and response rates of patients with HCC. Since the current body of evidence is moderate to very low, more robust randomized trials are needed to confirm the efficacy of HAIC.

**Systematic Review Registration:**

https://www.crd.york.ac.uk/prospero/display_record.php?RecordID=386780, identifier CRD42023386780.

## Introduction

The International Agency for Research on Cancer (IARC) reported that liver cancer was the sixth most common cancer and the third leading cause of cancer-related deaths worldwide in 2020 (1). In addition, the incidence of hepatocellular carcinoma (HCC) has increased over the past two decades. In the United States, the incidence of HCC is on the rise, particularly among individuals infected with the hepatitis C virus (HCV) (1, 2).

Current treatment strategies for HCC include surgical resection, transplantation, and locoregional therapies such as ablation, transarterial procedures, and systemic therapies. The treatment goals can vary based on the patient’s cancer stage, underlying liver function, and performance status. Consequently, numerous clinical practice guidelines for HCC recommend a multidisciplinary approach to developing individualized treatment plans (3–5). Usually, patients diagnosed with early-stage HCC are recommended for hepatic resection and liver transplantation. Systemic therapies, including tyrosine kinase inhibitors (TKIs) and immune checkpoint inhibitors, have achieved remarkable advances and are now recommended for patients with advanced HCC and distant metastasis. Intermediate or locally advanced stages of HCC encompass multifocal, diffuse, and infiltrative HCC. In some cases, patients with limited and well-defined lesions may be candidates for transplantation or transarterial chemoembolization. However, patients with extensive HCC liver involvement have limited therapeutic options (3). The Barcelona Clinic of Liver Cancer (BCLC) group and the National Comprehensive Cancer Network (NCCN) recommend systemic therapies as the primary approach for extensive disease (3, 6). While Asian groups, including groups in Korea, Japan, and China, suggest systemic therapies and hepatic arterial infusion chemotherapy (HAIC) as potential treatment options for these patients (4, 5, 7).

Several studies have demonstrated the effectiveness of cytotoxic chemotherapy on HCC (8, 9). However, accompanied liver cirrhosis affects the absorption and metabolism of chemotherapeutic agents, posing challenges in maintaining therapeutic doses and increasing the risk of toxicity. HAIC is a technique that involves the direct infusion of cytotoxic chemotherapy into the hepatic artery via an implanted catheter port system. This method is designed to expose HCC to high concentrations of chemotherapeutic agents while reducing adverse reactions (4, 10). Previous studies have shown favorable results of HAIC in patients with intermediate or advanced HCC (11–14). As a result, Eastern Asian groups such as the Korean Liver Cancer Association (KLCA) and the Japan Society of Hepatology (JSH) recommend HAIC as an important treatment option. In Korea, HAIC is recommended as a salvage therapy following the failure of first- or second-line systemic therapies, or as a substitute therapy for systemic therapies in advanced HCC patients with preserved liver function and portal vein invasion and without extrahepatic spread. In Japan, HAIC is considered for patients with more than four intrahepatic tumors or vascular invasion and who are not suitable candidates for local treatments, such as radiofrequency ablation (RFA) or transarterial chemoembolization (TACE) (4, 5). However, the effectiveness of HAIC compared to systemic therapy in patients is still controversial. Two randomized controlled trials (RCTs), the SILIUS study and the SCOOP-2 study, were conducted in Japan to compare the effectiveness of HAIC plus sorafenib with the sorafenib monotherapy (10, 15). While these studies did not find a significant difference in the overall survival between the two treatment groups, it is important to note that there were certain limitations in defining the impact of HAIC with these studies. Several meta-analyses and systematic reviews have been published, but most studies analyzed observational studies and RCTs together without distinction (16–18) and did not rate the certainty of evidence. Therefore, this study aimed to summarize the currently available evidence on the effects of HAIC with or without systemic chemotherapy versus systemic chemotherapy in patients with advanced HCC. This study was limited to RCTs, which provided a high certainty of evidence and used rigorous methodological standards for systematic review.

## Materials and methods

### Protocol registration and eligibility criteria

This study was conducted following the Preferred Reporting Items for Systematic Reviews and Meta-Analyses (PRISMA) guidelines and was based on a registered protocol (PROSPERO: CRD42023386780 from 14/01/2023). Institutional review board (IRB) approval was unnecessary for this type of study. The inclusion criteria for this systematic review and meta-analysis were RCTs involving patients with advanced HCC treated with HAIC, either with or without systemic chemotherapy. Trials in which the patients received HAIC as adjuvant therapy following surgical resection or in combination with RFA or TACE were excluded.

### Outcomes

The primary outcomes of this study were overall survival (OS) and progression-free survival (PFS). The secondary outcomes included adverse events (AEs), objective response rate (ORR), and disease control rate (DCR). ORR was defined as the sum of complete responses (CRs) and partial responses (PRs), and DCR was defined as the sum of CRs, PRs, and stable diseases. In all the included RCTs (10–15, 19), the modified Response Evaluation Criteria in Solid Tumors for HCC (mRECIST) was used to assess the treatment responses (20). AEs were assessed with the National Cancer Institute’s Common Terminology Criteria for Adverse Events (CTCAE).

### Search method for the identification and selection of studies

An experienced information specialist conducted the electric searches of multiple databases, including Medline via Ovid, Embase via Elsevier, the Cochrane Central Register of Controlled Trials via Wiley, Web of Science, ClinicalTrials.gov, WHO International Clinical Trials Registry Platform (ICTRP), Koreamed, and Kmbase. The search was performed from the inception of the databases to December 2022 without any language or publication status restrictions.

### Data extraction and risk of bias assessment

Two review authors (HJK, SHL) used the Covidence software platform (www.covidence.org) to assess all potentially relevant records and select eligible studies. Any discrepancies in the assessment of eligibility were resolved through discussions with a third member of the review team. After the eligible studies were selected, two review authors independently extracted data from each study in duplicate. The extracted data included study design, duration, setting, country, sample size, patient characteristics, interventions, clinical outcomes such as OS, PFS, DCR, and OCR, and adverse events. A PRISMA flow diagram is presented to illustrate the study selection process. The Cochrane Risk of bias tool was used to evaluate the risk of bias in each study (21). Each bias item was classified as ‘low risk,’ ‘high risk,’ or ‘unclear’.

### Data synthesis and analysis

Dichotomous data such as ORR, DCR, and adverse events are presented as risk ratios (RRs) with 95% confidence intervals (CIs). Time-to-events data, including OS and PFS, are expressed as hazard ratios (HRs) with 95% CIs. Heterogeneity (inconsistency) was identified by the *I* ^2^ statistics and interpreted according to the Cochrane Handbook (22). A visual inspection with Forest plots was used to assess the overlap of CIs. When any heterogeneities were detected, it was attempted to determine the possible reasons by evaluating the characteristics of each study and subgroup. A random-effects model was employed to summarize the data and interpret the results. The certainty of evidence (CoE) was rated on an outcome-specific basis using the Grading of Recommendations Assessment, Development, and Evaluation (GRADE) approach (23).

### Summary of the findings tables

To assess the overall quality of evidence, the GRADE framework was used. This framework considers criteria pertaining to validities, such as the risk of bias, imprecision, inconsistency, publication bias, and indirectness of results (23). Each author independently assigned a rating of high, moderate, low, or very low to the quality of evidence for each outcome in every comparison. Any inconsistencies were addressed through a consensus or, if necessary, by engaging other review authors for arbitration using GRADEpro (24). Subsequently, “Summary of findings” tables were generated that presented crucial information about the number of participants and studies, the certainty of the evidence, the estimated relative effects, and the anticipated absolute effects of each treatment strategy for each clinical outcome (25, 26). We used the GRADE guidance to describe the certainty of the evidence and the magnitude of the effect size (27).

### Analysis of subgroups

We planned to conduct subgroup analyses of the Child–Pugh score, level of tumor markers, and portal vein thrombosis and described them in the protocol. Additionally, subgroup analyses based on chemotherapy regimen were added to compare possible differences in the outcomes of each chemotherapeutic used for HAIC.

## Results

### Search results

**Figure 1:**
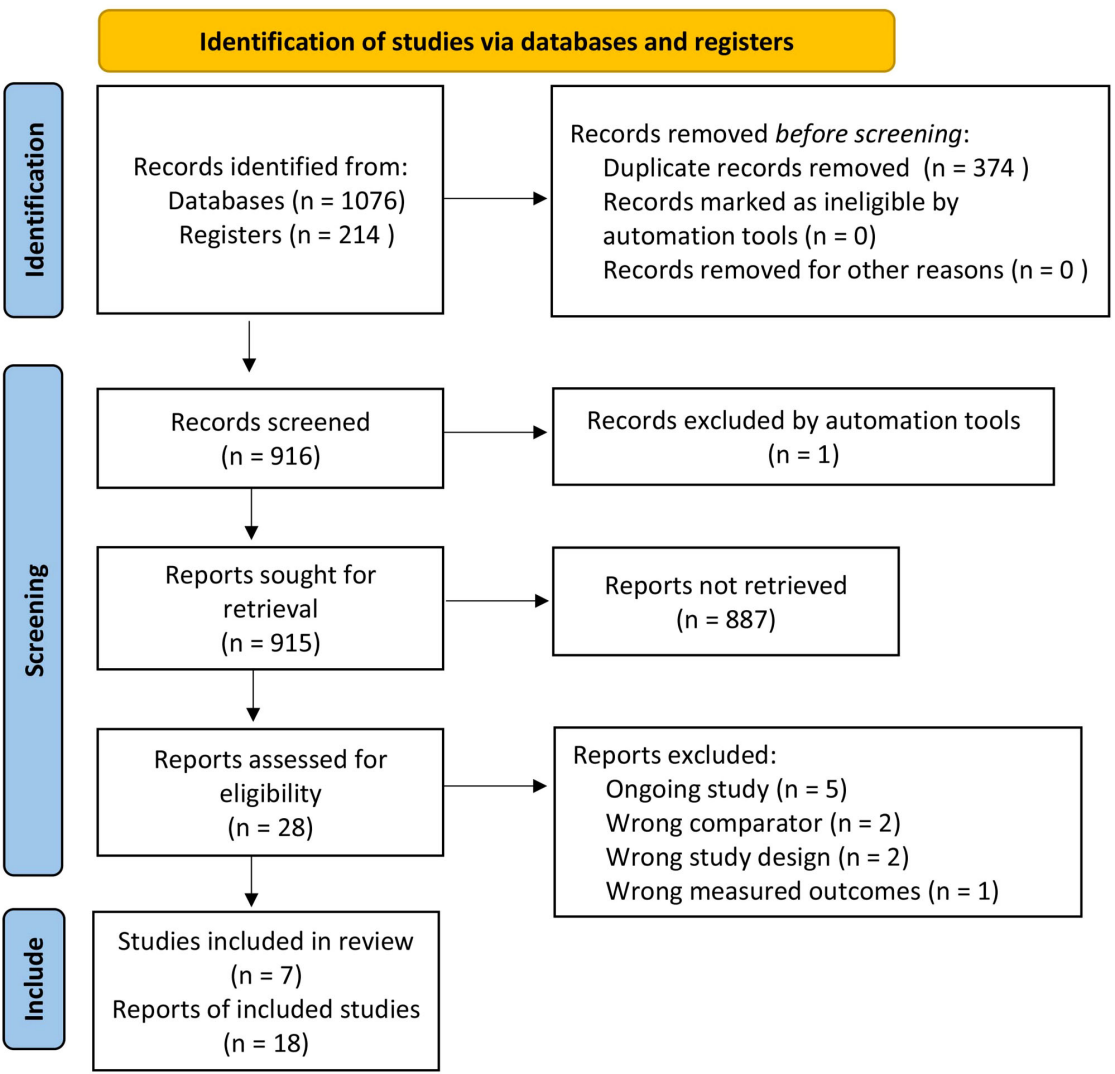
PRISMA flow diagram. PRISMA, Preferred Reporting Items for Systematic Reviews and Meta-Analyses.

**Figure 1** is the PRISMA flow diagram that presents the process of selecting eligible studies. A total of 1,076 records from databases and 214 records from registers were identified. After removing duplicates, 916 records were screened by the review authors. One record was excluded by automation tools, and 915 reports were sought for retrieval. Among them, 887 reports were not retrieved because of animal studies or exclusion criteria. Twenty-eight reports were assessed for eligibility. Five reports were excluded because they were ongoing studies and five other reports were excluded due to a wrong comparator (*n* = 2), wrong study design (*n* = 2), or wrong measured outcomes (*n* = 1). Finally, seven RCTs were included in this review (10–15, 19).

### Description of included studies

Among the seven RCTs included in this study, three studies were conducted in Japan (10, 15, 19), three in China (12–14), and one in South Korea (11). Five trials were multicenter trials (10, 11, 13, 15, 19), and the remaining two were single-center trials (12, 14). The included studies were performed between 2010 and 2020, and their publication dates ranged from 2016 to 2022. The number of eligible participants ranged from 58 to 262 individuals. Among the included RCTs, five trials compared HAIC + sorafenib and sorafenib alone, encompassing a total of 695 patients (10, 13–15, 19). The remaining two trials compared HAIC and sorafenib, with a total of 320 patients included (11, 12). In all the studies, the diagnosis of HCC was confirmed either histologically or clinically, according to the American Association for the Study of Liver disease criteria (2). All eligible patients were adults who were not suitable for surgery or locoregional treatment such as ablation or TACE (10–15, 19). Four trials included patients with ECOG PS 0–1 (10, 11, 15, 19), while the remaining three included patients with ECOG PS 0–2 (12–14). Two trials included patients with Child-Pugh grade A (Child-Pugh score 5 or 6) (13, 14), whereas the other five trials also included Child-Pugh score 7 (10–12, 15, 19). In all the studies, sorafenib was initiated at a dose of 400 mg twice daily (10–15, 19). However, there were variations in the HAIC regimens applied. Ikeda et al. and Kondo et al. utilized a cisplatin monotherapy as a HAIC regimen (15, 19), while Kudo et al. and Choi et al. used a combination of cisplatin and fluorouracil (10, 11). He et al., Zheng et al., and Lyu et al. employed a combination of oxaliplatin, leucovorin, and fluorouracil (12–14). All of the studies allowed for the participation of patients with extrahepatic metastasis as long as it was determined that the metastatic lesions would not have an influence on their prognosis (10–15, 19). One study only enrolled patients with portal vein invasion (13), while two studies exclusively included patients with major portal vein tumor thrombosis (Vp3–4) (11, 14). Although two studies initially used RECIST1.1 and conducted *post hoc* analyses with mRECIST (13, 14), all the included studies assessed tumor response using mRECIST. Six studies reported funding sources; five studies were supported by government agencies (10, 12–14, 19), and one study was funded by a non-profit organization (15). The other study did not specify its funding source (11). Five studies reported no conflicts of interest (11–14, 19), while two provided details of their conflicts of interest (10, 15). The characteristics of the included studies are summarized in **Supplement Table 1**.

### Risk of bias of the included studies

**Figure 2:**
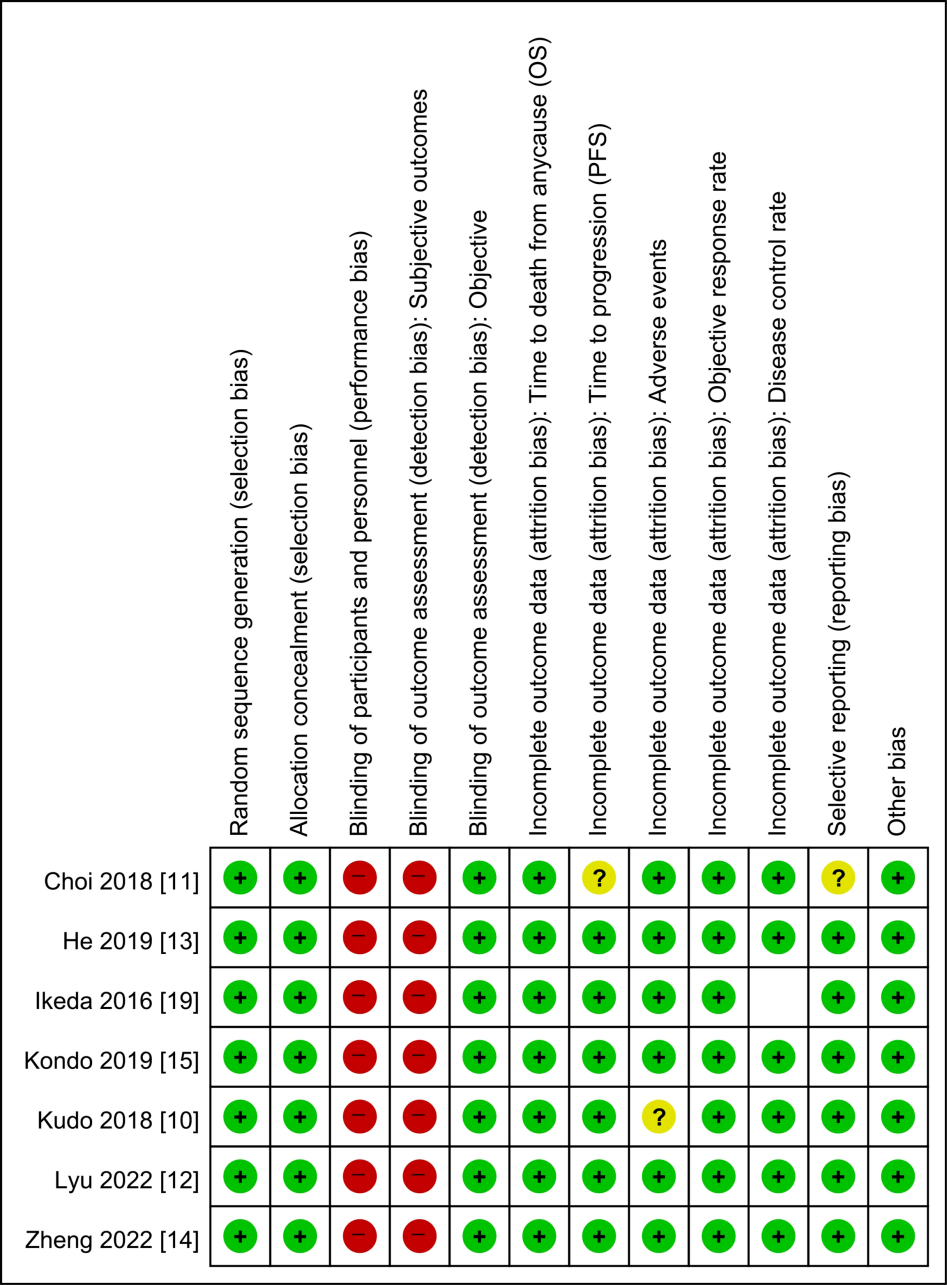
Risk of bias summary: review authors’ judgment on each risk of bias item for each induced study. OS, overall survival; PFS, progression-free survival.

The risks of bias in the included studies are summarized and presented in **Figure 2**. Most of the included studies had a low risk of bias across numerous domains; however, all the included studies were considered to have an overall high risk of performance bias and detection bias for the subjective outcomes. In the HAIC groups, patients had to undergo arterial catheterization, while the sorafenib groups did not. As a result, blinding of participants was inevitably impossible.

### Main analysis

#### Hepatic arterial infusion chemotherapy with sorafenib (HAIC + sorafenib) versus Sorafenib

Please refer to **Table 1**, and **Supplement Figures 1-6**.

**Table 1:** Summary of findings table of Hepatic arterial infusion chemotherapy + sorafenib versus sorafenib for advanced hepatocellular carcinoma.

Patient or population: unresectable hepatocellular carcinoma; Setting: Inpatients for Hepatic arterial infusion chemotherapy, Outpatients for sorafenib; Intervention: Hepatic arterial infusion chemotherapy (HAIC) + sorafenib; Comparison: Sorafenib					
Outcomes	No of participants (studies)	Certainty of the evidence (GRADE)	Relative effect (95% CI)	Anticipated absolute effects	
				Risk with Sorafenib	Risk difference with HAIC + sorafenib
**Overall survival** MCID: absolute 2% reduction/increase	690 (10, 13–15, 19)	⊕⊕⊕◯ Moderate^a,b,c^	**HR 0.57** (0.34 to 0.96)	200 per 1,000	**200 more per 1,000** (13 more to 379 more)
**Progression-free survival** MCID: absolute 5% reduction/increase	690 (10, 13–15, 19)	⊕⊕◯◯ Low^b,d,e^	**HR 0.56** (0.34 to 0.93)	300 per 1,000	**210 more per 1,000** (26 more to 364 more)
**Adverse events** assessed with: CTCAE version 4.0 MCID: absolute 5% reduction/increase	415 (13, 14, 19)	⊕⊕◯◯ Low^b,d,e^	**RR 1.02** (0.96 to 1.09)	918 per 1,000	**18 more per 1,000** (37 fewer to 83 more)
**Objective response rate** assessed with: mRECIST criteria MCID: absolute 5% reduction/increase	685 (10, 13–15, 19)	⊕⊕⊕◯ Moderate^b,d^	**RR 4.03** (1.68 to 9.63)	97 per 1,000	**293 more per 1,000** (66 more to 834 more)
**Disease control rate** assessed with: mRECIST criteria MCID: absolute 5% reduction/increase	584 (10, 13–15)	⊕◯◯◯ Very low^d,e,f^	**RR 1.39** (0.95 to 2.05)	566 per 1,000	**221 more per 1,000** (28 fewer to 594 more)
*The risk in the intervention group (and its 95% confidence interval) is based on the assumed risk in the comparison group and the **relative effect** of the intervention (and its 95% CI). **GRACE**: Grading of Recommendations Assessment, Development, and Evaluation; **CI:** confidence interval; **MCID**: minimal clinically important difference; **HR:** hazard Ratio; **RR:** risk ratio; **CTCAE**: National Cancer Institute’s Common Terminology Criteria for Adverse Events; **mRECIST**: modified Response Evaluation Criteria in Solid Tumors for HCC.					
**GRADE Working Group grades of evidence** **High certainty:** we are very confident that the true effect lies close to that of the estimate of the effect; **Moderate certainty:** we are moderately confident in the effect estimate: the true effect is likely to be close to the estimate of the effect, but there is a possibility that it is substantially different; **Low certainty:** our confidence in the effect estimate is limited: the true effect may be substantially different from the estimate of the effect; **Very low certainty:** we have very little confidence in the effect estimate: the true effect is likely to be substantially different from the estimate of effect					
**Explanations** a. Given the enrolled patient’s condition, the high risk of performance bias would not affect the overall survival; b. We did not downgrade for inconsistency because the heterogeneity of effect estimates resulted from chemo-regimen (cisplatin vs oxaliplatin); c. Confidence interval crosses assumed clinically important threshold (2%); d. High risk of detection bias; e. Confidence interval crosses assumed clinically important threshold (5%); f. Unexplained high heterogeneity *I* ^2 ^= 86%					

#### Primary outcomes

##### Overall survival

Five RCTs with 690 patients (HAIC + sorafenib *n* = 359, sorafenib *n* = 331) reported OS (10, 13–15, 19). HAIC + sorafenib probably increases OS compared to sorafenib (HR 0.57, 95% CI 0.34 to 0.96; *I* ^2 ^= 87%; moderate-certainty evidence). This corresponds to 200 more OS per 1000 patients (95% CI 13 more to 379 more) than sorafenib. We downgraded the certainty of the evidence for serious imprecision (–1). (**Table 1**; **Supplement Figure 1, 2**).

##### Progression-free survival

Five RCTs with 690 patients (HAIC + sorafenib *n* = 359, sorafenib *n* = 331) reported PFS (10, 13–15, 19). HAIC + sorafenib may increase PFS compared to sorafenib (HR 0.56, 95% CI 0.34 to 0.93; *I* ^2 ^= 96.8%; low-certainty evidence). This corresponds to 210 more PFS per 1000 patients (95% CI 26 more to 364 more) than sorafenib. We downgraded the certainty of the evidence for serious study limitations (–1) and serious imprecision (–1) (**Table 1**; **Supplement Figure 3**).

##### Adverse events

Three RCTs with 415 patients (HAIC + sorafenib *n* = 221, sorafenib *n* = 194) reported adverse events (13, 14, 19). HAIC + sorafenib may result in little to no difference in adverse events compared to sorafenib (RR 1.02, 95% CI 0.96 to 1.09; *I* ^2 ^= 46%; low-certainty evidence). This corresponds to 18 more adverse events per 1000 patients (95% CI 37 fewer to 83 more) than sorafenib. We downgraded the certainty of the evidence for serious study limitations (–1) and serious imprecision (–1). (**Table 1**; **Supplement Figure 4**).

#### Secondary outcomes

##### Objective response rate

Five RCTs with 685 patients (HAIC + sorafenib *n* = 354, sorafenib *n* = 331) reported ORR (10, 13–15, 19). HAIC + sorafenib probably increases ORR compared to sorafenib (RR 4.03, 95% CI 1.68 to 9.63; *I* ^2 ^= 75%; moderate-certainty evidence). This corresponds to 293 more ORR per 1000 patients (95% CI 66 more to 834 more) than sorafenib. We downgraded the certainty of the evidence for serious study limitations (–1) (**Table 1**; **Supplement Figure 5**).

##### Disease control rate

Four RCTs with 584 patients (HAIC + sorafenib *n* = 294, sorafenib *n* = 290) reported DCR (10, 13–15). HAIC + sorafenib may increase the disease control rate, but the evidence is very uncertain. (RR 1.39, 95% CI 0.95 to 2.05; *I* ^2 ^= 86%; very low-certainty evidence). We downgraded the certainty of the evidence for serious study limitations (–1), serious inconsistency (–1), and serious imprecision (–1) (**Table 1**; **Supplement Figure 6**).

#### Subgroup analyses

Subgroup analyses (stratified by chemo-regimen and portal vein thrombosis) on the primary outcomes were performed. Pre-planned subgroup analyses stratified by Child-Pugh scores and level of tumor markers could not be performed because there were no available data.

##### Chemo-regimen (cisplatin vs oxaliplatin)

In terms of OS and PFS, of the 690 patients, 379 were using cisplatin (HAIC + sorafenib *n* = 202; sorafenib *n* = 177), and 331 were using oxaliplatin (HAIC + sorafenib *n* = 157; sorafenib *n* = 154).

###### OS

For patients using cisplatin, the HR was 0.88 (95% CI 0.69 to 1.13), and for those using oxaliplatin, the HR was 0.34 (95% CI 0.26 to 0.44). The test for interaction was significant (*P* = 0.001, *I* ^2 ^= 96.3%) (**Supplement Figure 1**).

###### PFS

For patients using cisplatin, the HR was 0.81 (95% CI 0.65 to 1.00), and for those using oxaliplatin, the HR was 0.31 (95% CI 0.25 to 0.40). The test for interaction was significant (*P* = 0.001, *I* ^2 ^= 96.8%) (**Supplement Figure 3**).

###### Adverse events

Of the 415 patients; 106 were using cisplatin (HAIC + sorafenib *n* = 65; sorafenib *n* = 41), and 309 were using oxaliplatin (HAIC + sorafenib *n* = 156; sorafenib *n* = 153). For the patients using cisplatin, the RR was 1.00 (95% CI 0.96 to 1.04), and for those using oxaliplatin, the RR was 1.05 (95% CI 0.99 to 1.12). The test for interaction was not significant (*P* = 0.18, *I* ^2 ^= 43.3%) (**Supplement Figure 4**).

##### Portal vein thrombosis

###### OS

Of the 690 patients, 559 had portal vein thrombosis (HAIC + sorafenib *n* = 291; sorafenib *n* = 268), and 131 had no portal vein thrombosis (HAIC + sorafenib *n* = 68; sorafenib *n* = 63). For the patients with portal vein thrombosis, the HR was 0.58 (95% CI 0.32 to 1.05), and for those without portal vein thrombosis, the HR was 0.80 (95% CI 0.55 to 1.15). The test for interaction was not significant (*P* = 0.37, *I* ^2 ^= 0%) (**Supplement figure 2**).

###### Hepatic arterial infusion chemotherapy (HAIC) versus Sorafenib

Please refer to **Table 2**, and **Supplement Figures 7-11**.

**Table 2:** Summary of findings table of Hepatic arterial infusion chemotherapy versus sorafenib for advanced hepatocellular carcinoma.

Population: unresectable hepatocellular carcinoma Setting: Inpatients for Hepatic arterial infusion chemotherapy, Outpatients for sorafenib Intervention: Hepatic arterial infusion chemotherapy (HAIC) Comparison: Sorafenib					
Outcomes	No of participants (studies)	Certainty of the evidence (GRADE)	Relative effect (95% CI)	Anticipated absolute effects^*^ (95% CI)	
				Risk with Sorafenib	Risk difference with HAIC
**Overall survival** MCID: absolute 2% reduction/increase	320 (11, 12)	⊕⊕◯◯ Low^a,b^	**HR 0.42** (0.32 to 0.55)	250 per 1,000	**309 more per 1,000** (217 more to 392 more)
**Progression-free survival** MCID: absolute 5% reduction/increase	295 (11, 12)	⊕⊕◯◯ Low^b,c^	**HR 0.51** (0.38 to 0.69)	300 per 1,000	**241 more per 1,000** (136 more to 333 more)
**Adverse events** assessed with: CTCAE version 4.0 MCID: absolute 5% reduction/increase	320 (11, 12)	⊕⊕◯◯ Low^d,e^	**RR 0.93** (0.89 to 0.98)	988 per 1,000	**69 fewer per 1,000** (109 fewer to 20 fewer)
**Objective response rate** assessed with: mRECIST criteria MCID: absolute 5% reduction/increase	320 (11, 12)	⊕⊕◯◯ Low^b,d^	**RR 15.25** (4.84 to 48.10)	19 per 1,000	**266 more per 1,000** (72 more to 878 more)
**Disease control rate** assessed with: mRECIST criteria MCID: absolute 5% reduction/increase	320 (11, 12)	⊕◯◯◯ Very low^d,f,g^	**RR 1.85** (0.86 to 3.99)	528 per 1,000	**449 more per 1,000** (74 fewer to 1,579 more)
*The risk in the intervention group (and its 95% confidence interval) is based on the assumed risk in the comparison group and the **relative effect** of the intervention (and its 95% CI). **GRACE**: Grading of Recommendations Assessment, Development, and Evaluation; **CI:** confidence interval; **MCID**: minimal clinically important difference; **HR:** hazard Ratio; **RR:** risk ratio; **CTCAE**: National Cancer Institute’s Common Terminology Criteria for Adverse Events; **mRECIST**: modified Response Evaluation Criteria in Solid Tumors for HCC.					
**GRADE Working Group grades of evidence** **High certainty:** we are very confident that the true effect lies close to that of the estimate of the effect; **Moderate certainty:** we are moderately confident in the effect estimate: the true effect is likely to be close to the estimate of the effect, but there is a possibility that it is substantially different; **Low certainty:** our confidence in the effect estimate is limited: the true effect may be substantially different from the estimate of the effect; **Very low certainty:** we have very little confidence in the effect estimate: the true effect is likely to be substantially different from the estimate of effect					
**Explanations** a. Unclear risk of reporting bias; b. Small optimal information size; c. High risk of performance and detection bias and unclear risk of attrition and reporting bias; d. High risk of performance and detection bias and unclear risk of reporting bias; e. Confidence interval crosses assumed clinically important threshold (5%); f. Unexplained high heterogeneity *I* ^2 ^= 83%; g. We did not downgrade for imprecision because a wide confidence interval results from inconsistency					

#### Primary outcomes

##### Overall survival

Two RCTs with 320 patients (HAIC *n* = 159, sorafenib *n* = 161) reported OS (11, 12). HAIC may increase OS compared to sorafenib (HR 0.42, 95% CI 0.32 to 0.55; *I* ^2 ^= 0%; low-certainty evidence). This corresponds to 309 more OS per 1000 patients (95% CI 217 more to 392 more) than sorafenib. We downgraded the certainty of the evidence for serious study limitations (–1) and serious imprecision (–1) (**Table 2**; **Supplement Figure 7**).

##### Progression-free survival

Two RCTs with 295 patients (HAIC *n* = 144, sorafenib *n* = 151) reported PFS (11, 12). HAIC may increase PFS compared to sorafenib (HR 0.51, 95% CI 0.38 to 0.69; *I* ^2 ^= 39%; low-certainty evidence). This corresponds to 241 more PFS per 1000 patients (95% CI 136 more to 333 more) than sorafenib. We downgraded the certainty of the evidence for serious study limitations (–1) and serious imprecision (–1). (**Table 2**; **Supplement Figure 8**).

##### Adverse events

Two RCTs with 320 patients (HAIC *n* = 159, sorafenib *n* = 161) reported adverse events (11, 12). HAIC may reduce adverse events slightly compared to sorafenib (RR 0.93, 95% CI 0.89 to 0.98; *I* ^2 ^= 0%; low-certainty evidence). This corresponds to 69 fewer adverse events per 1000 patients (95% CI 109 fewer to 20 fewer) than sorafenib. We downgraded the certainty of the evidence for serious study limitations (–1) and serious imprecision (–1) (**Table 2**; **Supplement Figure 9**).

#### Secondary outcomes

##### Objective response rate

Two RCTs with 320 patients (HAIC *n* = 159, sorafenib *n* = 161) reported ORR (11, 12). HAIC may increase ORR compared to sorafenib (RR 15.25, 95% CI 4.84 to 48.10; *I* ^2 ^= 0%; low-certainty evidence). This corresponds to 266 more ORR per 1000 patients (95% CI 72 more to 878 more) than sorafenib. We downgraded the certainty of the evidence for serious study limitations (–1) and serious imprecision (–1) (**Table 2**; **Supplement Figure 10**).

##### Disease control rate

Two RCTs with 320 patients (HAIC *n* = 159, sorafenib *n* = 161) reported DCR (11, 12). HAIC may increase the disease control rate, but the evidence is very uncertain (RR 1.85, 95% CI 0.86 to 3.99; *I* ^2 ^= 83%; very low-certainty evidence). We downgraded the certainty of the evidence for serious study limitations (–1), serious inconsistency (–1), and serious imprecision (–1) (**Table 2**; **Supplement Figure 11**).

#### Subgroup analyses

Subgroup analyses (stratified by chemo-regimen) on the primary outcomes were performed. Pre-planned subgroup analyses stratified by portal vein thrombosis, Child-Pugh scores, and level of tumor markers could not be performed because there were no available data.

##### Chemo-regimen (cisplatin vs oxaliplatin)

Regarding OS and adverse events, of the 320 patients, 58 were using cisplatin (HAIC *n* = 29; sorafenib *n* = 29), and 162 were using oxaliplatin (HAIC *n* = 130; sorafenib *n* = 132).

###### OS

For the patients using cisplatin, the HR was 0.48 (95% CI 0.27 to 0.85), and for those using oxaliplatin, the HR was 0.41 (95% CI 0.30 to 0.55). The test for interaction was not significant (*P* = 0.62, *I* ^2 ^= 0%) (**Supplement Figure 7**).

###### PFS

Of the 295 patients; 33 were using cisplatin (HAIC *n* = 14; sorafenib *n* = 19), and 162 were using oxaliplatin (HAIC *n* = 130; sorafenib *n* = 132). For the patients using cisplatin, the HR was 0.61 (95% CI 0.42 to 0.90), and for those using oxaliplatin, the HR was 0.45 (95% CI 0.34 to 0.60). The test for interaction was not significant (*P* = 0.20, *I* ^2 ^= 38.8%) (**Supplement Figure 8**).

###### Adverse events

For the patients using cisplatin, the RR was 0.93 (95% CI 0.78 to 1.10), and for those using oxaliplatin, the RR was 0.93 (95% CI 0.89 to 0.98). The test for interaction was not significant (*P* = 0.95, *I* ^2 ^= 0%) (**Supplement Figure 9**).

## Discussion

### Main findings

Seven RCTs comprising 1,010 patients that compared sorafenib monotherapy with either HAIC + sorafenib or HAIC alone were identified (10–15, 19). The findings of this meta-analysis study suggest that HAIC, with or without sorafenib, may improve OS, PFS, and response rates in advanced HCC patients without significant differences in adverse events.

Most of the included RCTs demonstrated the survival benefits of HAIC treatments, while the studies conducted by Kondo et al. and Kudo et al. did not show such benefits (10, 15). However, it is imperative to approach the interpretation of their results with caution. Both trials utilized cisplatin monotherapy or low-dose cisplatin with fluorouracil. In the study by Kondo et al., the concept of “Clinical PD” was introduced based on the levels of alpha-fetoprotein (AFP) and des-gamma carboxyprothrombin (DCP). However, the authors interpreted that clinical PD is unlikely to be disadvantageous. Furthermore, in the sorafenib group, more than half of the patients who received subsequent treatments underwent HAIC, suggesting a potential crossover effect that should not be overlooked (15). In the study by Kudo et al., the calculated sample size for each group was 95 patients, and 102 patients were allocated to the HAIC + sorafenib group. However, 14 patients did not receive treatment, resulting in an underpowered sample size (10).

The initial expectation was that the combination of sorafenib and HAIC would yield better results compared to HAIC alone. Overall, HAIC plus sorafenib showed favorable OS, PFS, and ORRs compared with sorafenib alone; however, those of the HAIC alone groups were numerically better (**Tables 1**, **2**). Due to significant heterogeneities observed in the analyses of HAIC plus sorafenib, conducting subgroup analyses based on the HAIC regimen provided valuable insights into the underlying reasons (**Supplement Figures 1, 3**). Among the five RCTs that used HAIC plus sorafenib, three utilized cisplatin-based chemotherapy (10, 15, 19), while the other two employed oxaliplatin-based chemotherapy (13, 14). As depicted in **Supplement Figures 1 and 3**, the subgroup receiving the oxaliplatin-based treatment demonstrated significantly better survival outcomes compared with the subgroup receiving the cisplatin-based treatment. The two RCTs investigating HAIC alone employed a higher dose of cisplatin (60 mg/m^2^/cycle) or oxaliplatin in combination with fluorouracil (11, 12). Additionally, notable differences were observed in the intervals between each HAIC cycle. In the subgroup receiving the cisplatin-based chemotherapy, the intervals ranged from 4 to 6 weeks (10, 15, 19), which were longer than the intervals in the other studies, which ranged from 3 to 4 weeks (11–14).

In all the included RCTs, patients treated with HAIC demonstrated higher response rates than patients receiving sorafenib (range, HAIC with or without sorafenib vs. sorafenib, 17.1–54.8% vs. 3.1–18.0%). Particularly, He et al. (13). reported that a significantly higher number of patients in the HAIC plus sorafenib group proceeded to curative surgery compared with the sorafenib group (HAIC + sorafenib vs. sorafenib, 16 [12.8%] vs. 1 [0.8%], *P* < 0.001). Similar results were reported by Lyu et al. (12). These findings suggest that HAIC may contribute to downstaging and enabling a switch to curative surgery, thereby improving survival outcomes. In the phase III trial of sorafenib conducted in Western countries, the ORR of sorafenib was 2% (7 of 299 patients), and in the Asia-Pacific region, the ORR was 3.3% (5 of 150 patients) (28, 29). In the updated report of the IMbrave150 study, the ORR based on mRECIST for atezolizumab plus bevacizumab was 30% (97 of 326 patients), while the ORR for sorafenib was 11% (18 of 159 patients) (30). Considering the relatively high ORR observed with HAIC and the possibility of crossover to curative resection or locoregional therapy, HAIC may have a crucial role in the treatment of HCC, even in the era of immune checkpoint inhibitors.

Major vascular invasion or portal vein thrombosis are significant adverse prognostic factors for HCC (3). In the subgroup analyses according to the portal vein invasion and thrombosis (PVTT), HAIC plus sorafenib showed a trend toward improved OS and PFS compared to sorafenib alone, especially in patients with PVTT. However, it did not reach statistical significance.

### Relation to previous works

There have been several published systematic reviews on this topic that have presented the positive effect of HAIC on HCC treatments. Long et al. recently reported that sorafenib plus HAIC showed significantly better OS (HR 0.56 [95% CI 0.37–0.83]; *P* < 0.01), PFS (HR 0.44 [95% CI 0.27–0.72]; *P* < 0.01), and ORR (RR 3.77 [95% CI 1.87–7.58]; *P* < 0.01) than sorafenib alone (31). Zhang et al. also conducted a recent systematic review, comparing HAIC to sorafenib in advanced HCC with PVTT, and reported significant improvements in OS (HR 0.50, 95% CI 0.40–0.63, *P* < 0.05), PFS (HR = 0.49, 95% CI 0.35–0.67, *P* < 0.05), and ORR (RR 4.21, 95% CI 2.44–7.28, *P* < 0.000001) (32). Additionally, this study demonstrated the benefits of HAIC, whether with or without sorafenib.

However, prior reported systematic reviews have exhibited some methodologic errors. Two systematic reviews were published in 2019 and 2022. Although the authors assessed the quality of the studies, they did not evaluate the risk of bias in the included studies (16, 33). Zhang et al. evaluated the quality of the cohort studies using the Newcastle Ottawa scale and conducted a risk assessment according to the Cochrane Collaboration Network recommendations (32). However, they also neglected to include the risk of bias assessment. Long et al. used the GRADE method and presented the risk of bias but did not incorporate this into the interpretation of the review results (31). However, the most considerable error is that all the aforementioned systematic reviews conducted meta-analyses by combining retrospective or observational studies with prospective RCTs. These methodologic errors can introduce flaws in the results.

### Strength and limitations

The rigorous methodology is a strength of this review. This systematic review was conducted based on a prospectively registered protocol, and an experienced information specialist performed the comprehensive literature search. Unlike most of the previous reviews, this study exclusively included RCTs for the meta-analysis. Furthermore, this review is unique in that the GRADE method was adopted, incorporating a certainty of evidence rating and presenting the absolute effect sizes within a clinical context.

However, this study also has some limitations. The combination of atezolizumab with bevacizumab is currently the first choice for advanced HCC, and other targeted agents, such as Lenvatinib, are also an acceptable alternative treatment for advanced HCC. However, since there were no RCTs comparing HAIC to these systemic therapies, we were unable to investigate the relative impacts of HAIC compared to the recently updated treatment for HCC. All of the included RCTs were performed in Asian countries, where the main cause of HCC is hepatitis B virus (HBV) infections. In contrast, patients with hepatitis C virus (HCV) infections, which are the primary cause of HCC in Western countries, comprised a small portion of this study. Therefore, it is necessary to conduct more RCTs in Western countries to obtain a comprehensive understanding of the effectiveness of HAIC for HCC treatment across different populations and etiologies. The small sample size of RCTs was another limitation of this study. Since the current body of evidence is moderate to very low, more robust randomized trials are needed to confirm the efficacy of HAIC.

### Implications

This study provides evidence supporting the use of HAIC to treat advanced HCC. Furthermore, this study suggests that HAIC with an oxaliplatin-based regimen may contribute to a higher survival rate than HAIC with a cisplatin-based regimen.

HAIC represents a potential alternative for advanced HCC treatment, offering advantages over systemic therapies. Combinations of cytotoxic chemotherapy and targeted agents have emerged as a major trend in anti-cancer treatments, but their application in HCC has been limited due to the low efficacy of systemic administration of cytotoxic chemotherapy. HAIC offers the possibility of combining therapies in HCC while minimizing systemic adverse events. Additionally, HAIC may serve as a bridging therapy or induction therapy for curative resection or locoregional treatments.

## Data availability statement

The original contributions presented in the study are included in the article/**Supplementary Material**. Further inquiries can be directed to the corresponding authors.

## Author contributions

HK: Data curation, Formal Analysis, Investigation, Methodology, Project administration, Validation, Visualization, Writing – original draft, Writing – review & editing. SL: Data curation, Formal Analysis, Investigation, Methodology, Project administration, Validation, Visualization, Writing – original draft, Writing – review & editing. HS: Investigation, Methodology, Supervision, Validation, Writing – review & editing. HB: Formal Analysis, Investigation, Methodology, Writing – original draft. SC: Investigation, Methodology, Supervision, Validation, Writing – review & editing. IC: Conceptualization, Investigation, Methodology, Supervision, Validation, Writing – review & editing. EH: Formal Analysis, Investigation, Methodology, Software, Supervision, Visualization, Writing – review & editing. JH: Conceptualization, Data curation, Formal Analysis, Investigation, Methodology, Project administration, Resources, Supervision, Writing – review & editing, Writing – original draft. WB: Conceptualization, Data curation, Formal Analysis, Funding acquisition, Investigation, Methodology, Project administration, Resources, Supervision, Writing – review & editing, Writing – original draft.
