# Supplementary material for: Hepatic arterial infusion chemotherapy versus systemic therapy for advanced hepatocellular carcinoma: a systematic review and meta-analysis
Source: Front Oncol. 2023 Oct 10;13:1265240. doi: 10.3389/fonc.2023.1265240 (PMC10597692; doi:10.3389/fonc.2023.1265240)

- Figure1. HAIC + sorafenib vs Sorafenib; Overall survival according to the chemo-regimen

Study or Subgroup	log[Hazard Ratio]	SE	HAIC + sorafenib Total	Sorafenib Total	Weight	Hazard Ratio IV, Random, 95% CI
**1.1.1 Chemo-regimen (Cisplatin)**						
Ikeda 2016 [19]	-0.3857	0.2221	65	41	20.6%	0.68 [0.44, 1.05]
Kondo 2019 [15]	-0.1393	0.3596	35	33	16.8%	0.87 [0.43, 1.76]
Kudo 2018 [10]	0.009	0.1561	102	103	22.2%	1.01 [0.74, 1.37]
**Subtotal (95% CI)**			**202**	**177**	**59.7%**	**0.88 [0.69, 1.13]**
Heterogeneity: $Tau^2 = 0.00$; $Chi^2 = 2.12$, df = 2 (P = 0.35); $I^2 = 5\%$						
Test for overall effect: Z = 1.02 (P = 0.31)						
**1.1.2 Chemo-regimen (Oxaliplatin)**						
He 2019 [13]	-1.0498	0.1517	125	122	22.3%	0.35 [0.26, 0.47]
Zheng 2022 [14]	-1.273	0.3185	32	32	18.0%	0.28 [0.15, 0.52]
**Subtotal (95% CI)**			**157**	**154**	**40.3%**	**0.34 [0.26, 0.44]**
Heterogeneity: $Tau^2 = 0.00$; $Chi^2 = 0.40$, df = 1 (P = 0.53); $I^2 = 0\%$						
Test for overall effect: Z = 7.97 (P < 0.00001)						
**Total (95% CI)**			**359**	**331**	**100.0%**	**0.57 [0.34, 0.96]**
Heterogeneity: $Tau^2 = 0.30$; $Chi^2 = 30.68$, df = 4 (P < 0.00001); $I^2 = 87\%$						
Test for overall effect: Z = 2.10 (P = 0.04)						
Test for subgroup differences: $Chi^2 = 26.79$, df = 1 (P < 0.00001), $I^2 = 96.3\%$						

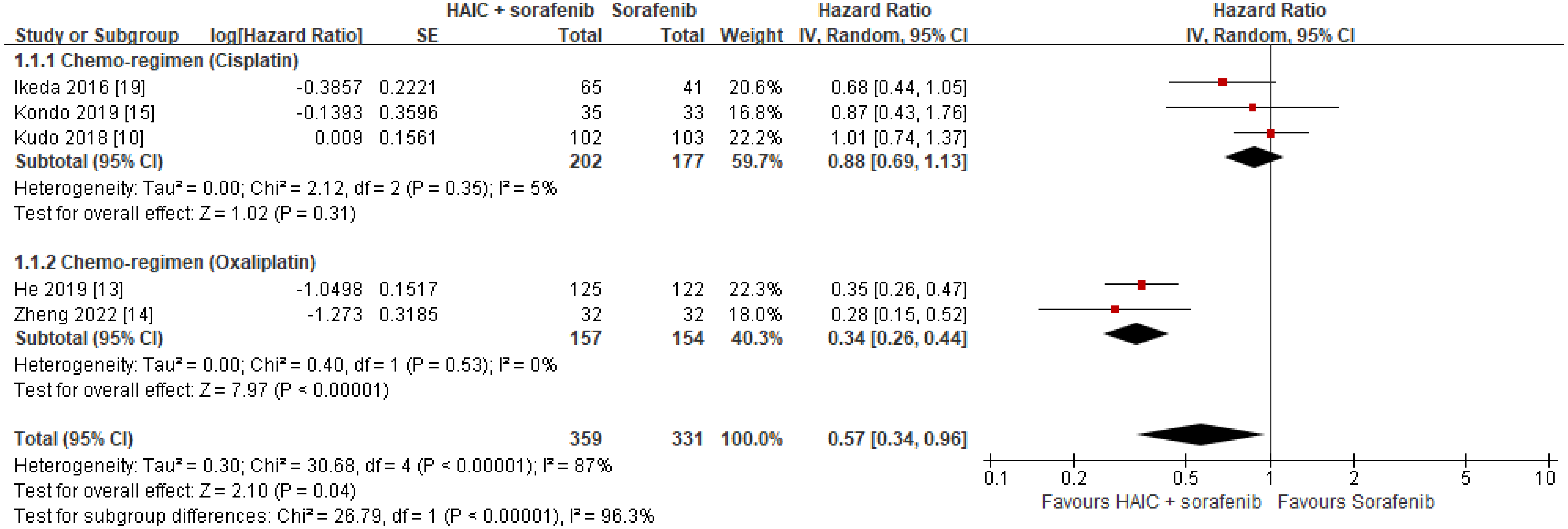

- Figure 2. HAIC + sorafenib vs Sorafenib; Overall survival according to the portal vein thrombosis

Study or Subgroup	log[Hazard Ratio]	SE	HAIC + sorafenib Total	Sorafenib Total	Weight	Hazard Ratio IV, Random, 95% CI
**1.2.1 Portal vein thrombosis (+)**						
He 2019 [13]	-1.0498	0.1517	125	122	15.5%	0.35 [0.26, 0.47]
Ikeda 2016 [19]	-0.5465	0.322	40	17	12.1%	0.58 [0.31, 1.09]
Kondo 2019 [15]	0.27	0.4522	21	22	9.4%	1.31 [0.54, 3.18]
Kudo 2018 [10]	0.0344	0.1866	73	75	14.9%	1.03 [0.72, 1.49]
Zheng 2022 [14]	-1.273	0.3185	32	32	12.1%	0.28 [0.15, 0.52]
**Subtotal (95% CI)**			**291**	**268**	**64.0%**	**0.58 [0.32, 1.05]**
Heterogeneity: Tau² = 0.37; Chi² = 28.44, df = 4 (P < 0.0001); I² = 86%						
Test for overall effect: Z = 1.80 (P = 0.07)						
**1.2.2 Portal vein thrombosis (-)**						
Ikeda 2016 [19]	-0.437	0.3245	25	24	12.0%	0.65 [0.34, 1.22]
Kondo 2019 [15]	-0.1393	0.3596	14	11	11.3%	0.87 [0.43, 1.76]
Kudo 2018 [10]	-0.1177	0.2926	29	28	12.7%	0.89 [0.50, 1.58]
**Subtotal (95% CI)**			**68**	**63**	**36.0%**	**0.80 [0.55, 1.15]**
Heterogeneity: Tau² = 0.00; Chi² = 0.62, df = 2 (P = 0.73); I² = 0%						
Test for overall effect: Z = 1.23 (P = 0.22)						
**Total (95% CI)**			**359**	**331**	**100.0%**	**0.65 [0.43, 0.97]**
Heterogeneity: Tau² = 0.26; Chi² = 32.70, df = 7 (P < 0.0001); I² = 79%						
Test for overall effect: Z = 2.09 (P = 0.04)						
Test for subgroup differences: Chi² = 0.79, df = 1 (P = 0.37), I² = 0%						

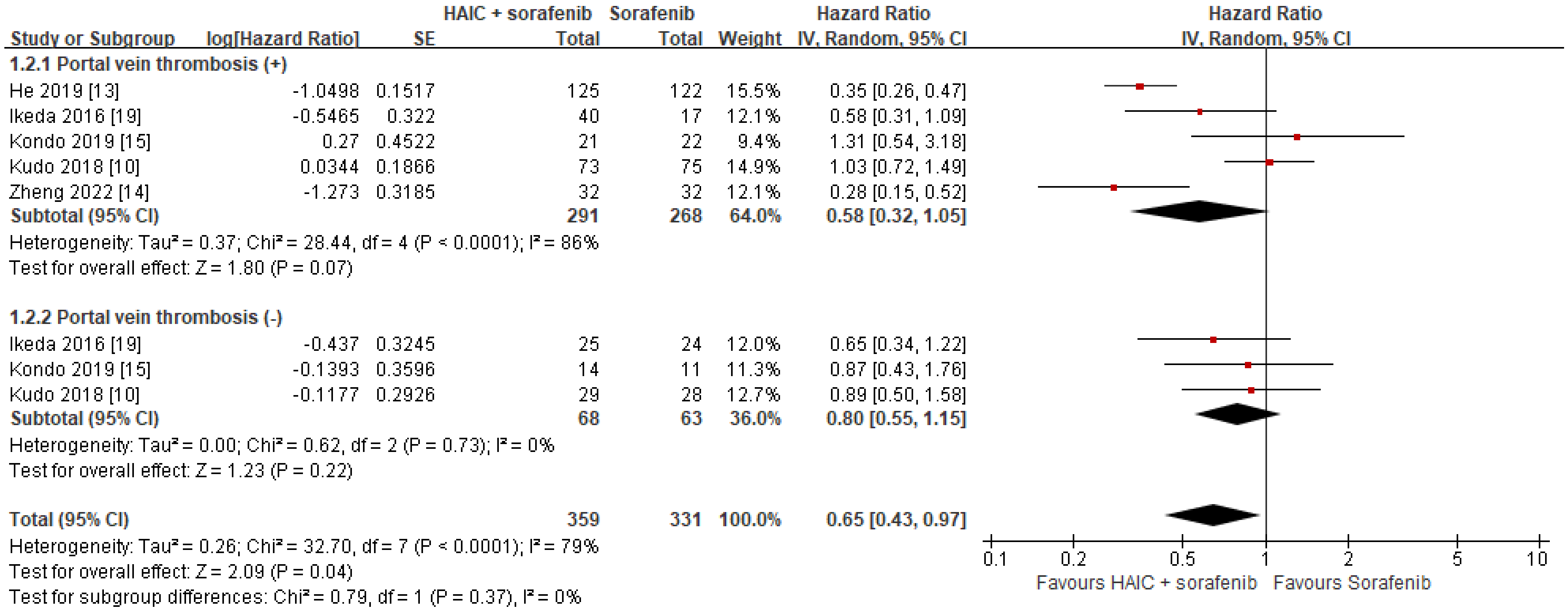

- Figure3. HAIC + sorafenib vs Sorafenib; Progression-free survival according to the chemo-regimen

Study or Subgroup	log[Hazard Ratio]	SE	HAIC + sorafenib Total	Sorafenib Total	Weight	Hazard Ratio IV, Random, 95% CI
**1.3.1 Chemo-regimen (Cisplatin)**						
Ikeda 2016 [19]	-0.2485	0.2069	65	41	20.3%	0.78 [0.52, 1.17]
Kondo 2019 [15]	0.157	0.2999	35	33	17.8%	1.17 [0.65, 2.11]
Kudo 2018 [10]	-0.2837	0.1457	102	103	21.8%	0.75 [0.57, 1.00]
**Subtotal (95% CI)**			**202**	**177**	**59.8%**	**0.81 [0.65, 1.00]**
Heterogeneity: Tau² = 0.00; Chi² = 1.79, df = 2 (P = 0.41); I² = 0%						
Test for overall effect: Z = 1.93 (P = 0.05)						
**1.3.2 Chemo-regimen (Oxaliplatin)**						
He 2019 [13]	-1.1087	0.1417	125	122	21.9%	0.33 [0.25, 0.44]
Zheng 2022 [14]	-1.3471	0.2806	32	32	18.3%	0.26 [0.15, 0.45]
**Subtotal (95% CI)**			**157**	**154**	**40.2%**	**0.31 [0.25, 0.40]**
Heterogeneity: Tau² = 0.00; Chi² = 0.58, df = 1 (P = 0.45); I² = 0%						
Test for overall effect: Z = 9.15 (P < 0.00001)						
**Total (95% CI)**			**359**	**331**	**100.0%**	**0.56 [0.34, 0.93]**
Heterogeneity: Tau² = 0.28; Chi² = 33.87, df = 4 (P < 0.00001); I² = 88%						
Test for overall effect: Z = 2.23 (P = 0.03)						
Test for subgroup differences: Chi² = 31.51, df = 1 (P < 0.00001), I² = 96.8%						

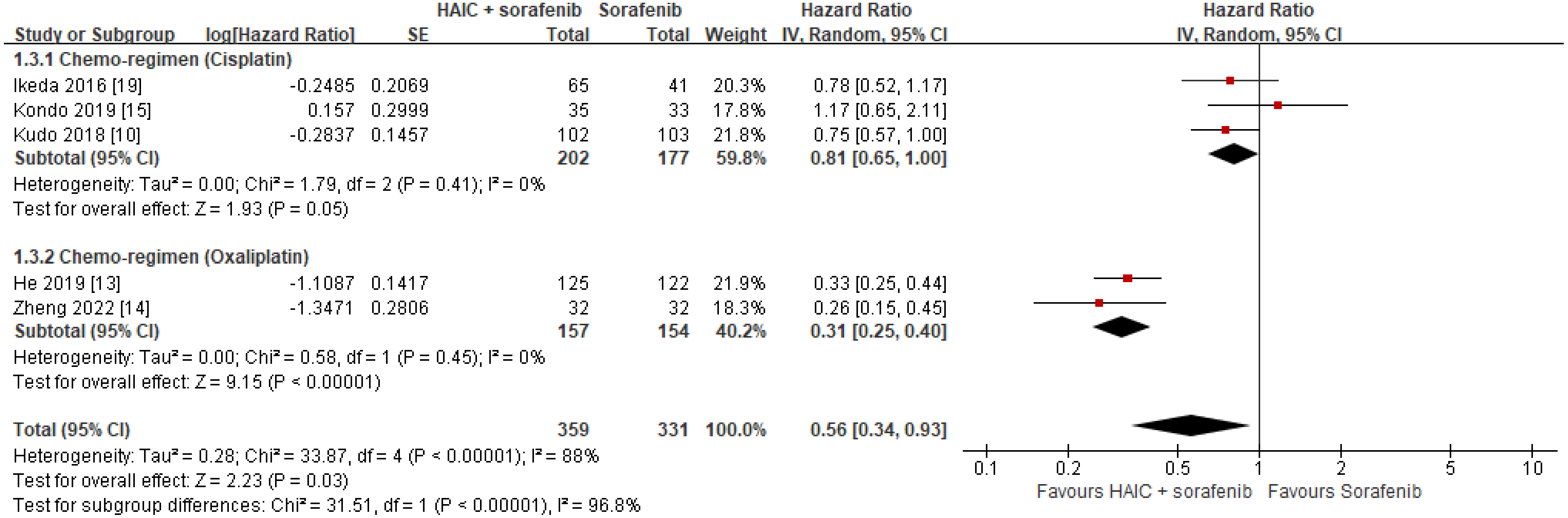

- Figure 4. HAIC + sorafenib vs Sorafenib; Adverse events according to the chemo-regimen

Study or Subgroup	HAIC + sorafenib Events	HAIC + sorafenib Total	Sorafenib Events	Sorafenib Total	Weight	Risk Ratio M-H, Random, 95% CI
**1.4.1 Chemo-regimen (Cisplatin)**						
Ikeda 2016 [19]	65	65	41	41	54.7%	1.00 [0.96, 1.04]
**Subtotal (95% CI)**		**65**		**41**	**54.7%**	**1.00 [0.96, 1.04]**
Total events	65		41			
Heterogeneity: Not applicable						
Test for overall effect: Z = 0.00 (P = 1.00)						
**1.4.2 Chemo-regimen (Oxaliplatin)**						
He 2019 [13]	118	124	109	121	35.2%	1.06 [0.98, 1.13]
Zheng 2022 [14]	29	32	28	32	10.1%	1.04 [0.87, 1.23]
**Subtotal (95% CI)**		**156**		**153**	**45.3%**	**1.05 [0.99, 1.12]**
Total events	147		137			
Heterogeneity: Tau² = 0.00; Chi² = 0.04, df = 1 (P = 0.83); I² = 0%						
Test for overall effect: Z = 1.55 (P = 0.12)						
**Total (95% CI)**		**221**		**194**	**100.0%**	**1.02 [0.96, 1.09]**
Total events	212		178			
Heterogeneity: Tau² = 0.00; Chi² = 3.72, df = 2 (P = 0.16); I² = 46%						
Test for overall effect: Z = 0.76 (P = 0.45)						
Test for subgroup differences: Chi² = 1.77, df = 1 (P = 0.18), I² = 43.3%						

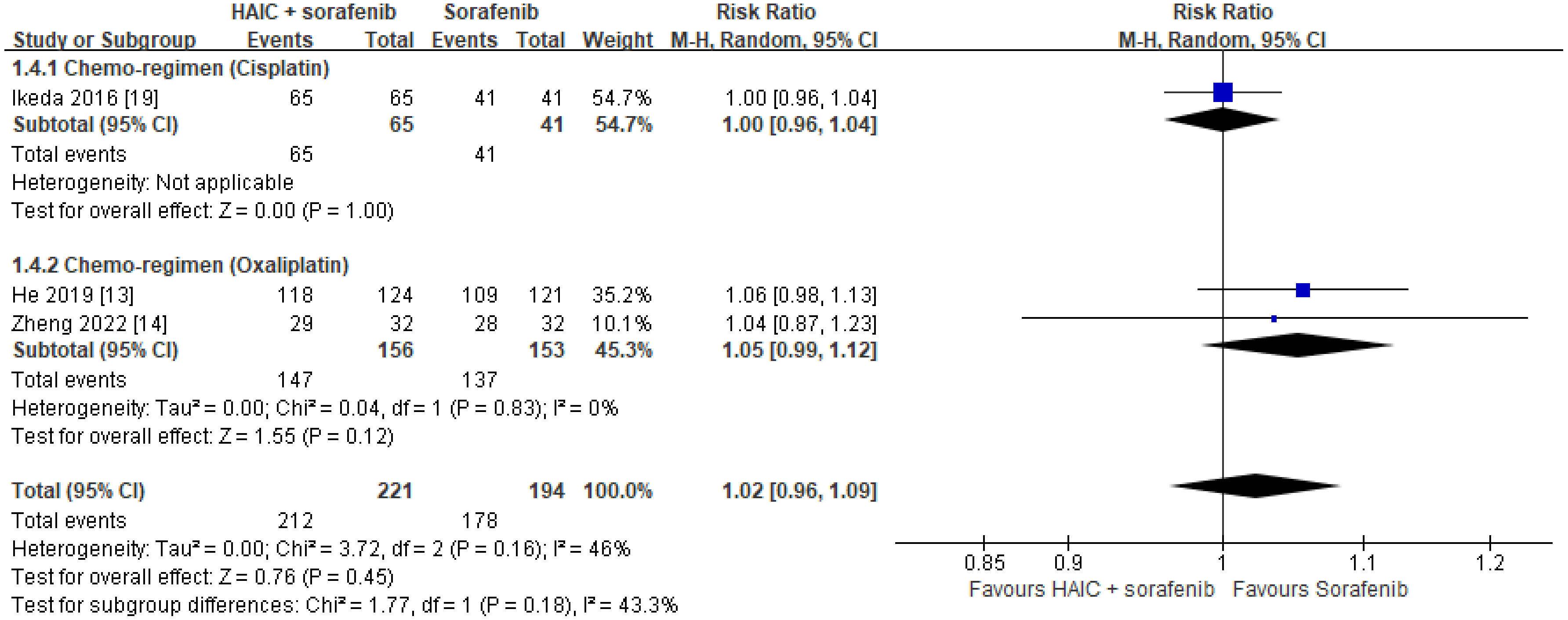

- Figure 5. HAIC + sorafenib vs Sorafenib; Objective response rate

	HAIC + sorafenib		Sorafenib			Risk Ratio
Study or Subgroup	Events	Total	Events	Total	Weight	M-H, Random, 95% CI
He 2019 [13]	68	125	7	122	24.4%	9.48 [4.54, 19.81]
Ikeda 2016 [19]	13	60	3	41	19.0%	2.96 [0.90, 9.74]
Kondo 2019 [15]	6	35	3	33	17.8%	1.89 [0.51, 6.93]
Kudo 2018 [10]	37	102	18	103	27.0%	2.08 [1.27, 3.40]
Zheng 2022 [14]	16	32	1	32	11.8%	16.00 [2.25, 113.59]
**Total (95% CI)**		**354**		**331**	**100.0%**	**4.03 [1.68, 9.63]**
Total events	140		32			

Heterogeneity: Tau² = 0.67; Chi² = 16.10, df = 4 (P = 0.003); I² = 75%
Test for overall effect: Z = 3.13 (P = 0.002)

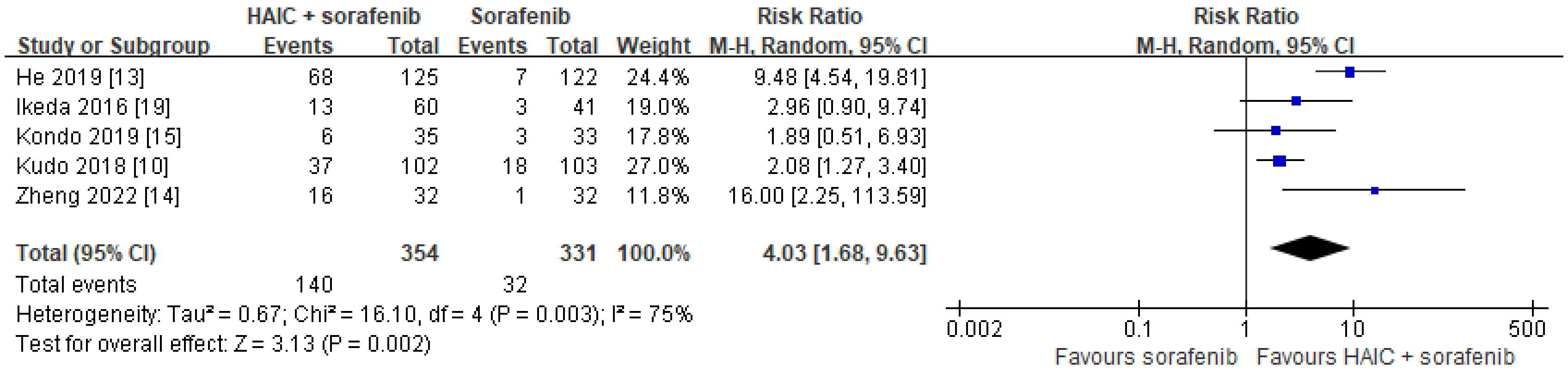

- Figure 6. HAIC + sorafenib vs Sorafenib; Disease control rate

Study or Subgroup	HAIC + sorafenib Events	HAIC + sorafenib Total	Sorafenib Events	Sorafenib Total	Weight	Risk Ratio M-H, Random, 95% CI
He 2019 [13]	95	125	62	122	28.4%	1.50 [1.22, 1.83]
Kondo 2019 [15]	24	35	15	33	22.1%	1.51 [0.98, 2.33]
Kudo 2018 [10]	66	102	75	103	28.7%	0.89 [0.74, 1.07]
Zheng 2022 [14]	26	32	12	32	20.9%	2.17 [1.34, 3.49]
**Total (95% CI)**		**294**		**290**	**100.0%**	**1.39 [0.95, 2.05]**
Total events	211		164			

Heterogeneity: $Tau^2 = 0.13$; $Chi^2 = 22.19$, $df = 3$ ($P < 0.0001$); $I^2 = 86\%$

Test for overall effect: $Z = 1.69$ ($P = 0.09$)

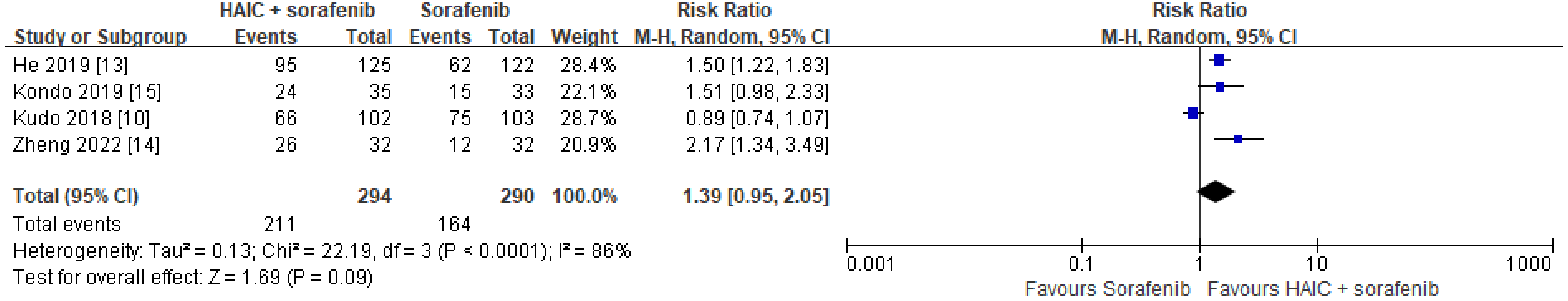

- Figure 7. HAIC vs Sorafenib; Overall survival

Study or Subgroup	log[Hazard Ratio]	SE	HAIC Total	Sorafenib Total	Weight	Hazard Ratio IV, Random, 95% CI
**2.1.1 Chemo-regimen (Cisplatin)**						
Choi 2018 [11]	-0.734	0.2936	29	29	21.8%	0.48 [0.27, 0.85]
**Subtotal (95% CI)**			**29**	**29**	**21.8%**	**0.48 [0.27, 0.85]**
Heterogeneity: Not applicable						
Test for overall effect: Z = 2.50 (P = 0.01)						
**2.1.2 Chemo-regimen (Oxaliplatin)**						
Lyu 2022 [12]	-0.8965	0.1552	130	132	78.2%	0.41 [0.30, 0.55]
**Subtotal (95% CI)**			**130**	**132**	**78.2%**	**0.41 [0.30, 0.55]**
Heterogeneity: Not applicable						
Test for overall effect: Z = 5.78 (P < 0.00001)						
**Total (95% CI)**			**159**	**161**	**100.0%**	**0.42 [0.32, 0.55]**

Heterogeneity: Tau² = 0.00; Chi² = 0.24, df = 1 (P = 0.62); I² = 0%

Test for overall effect: Z = 6.28 (P < 0.00001)

Test for subgroup differences: Chi² = 0.24, df = 1 (P = 0.62), I² = 0%

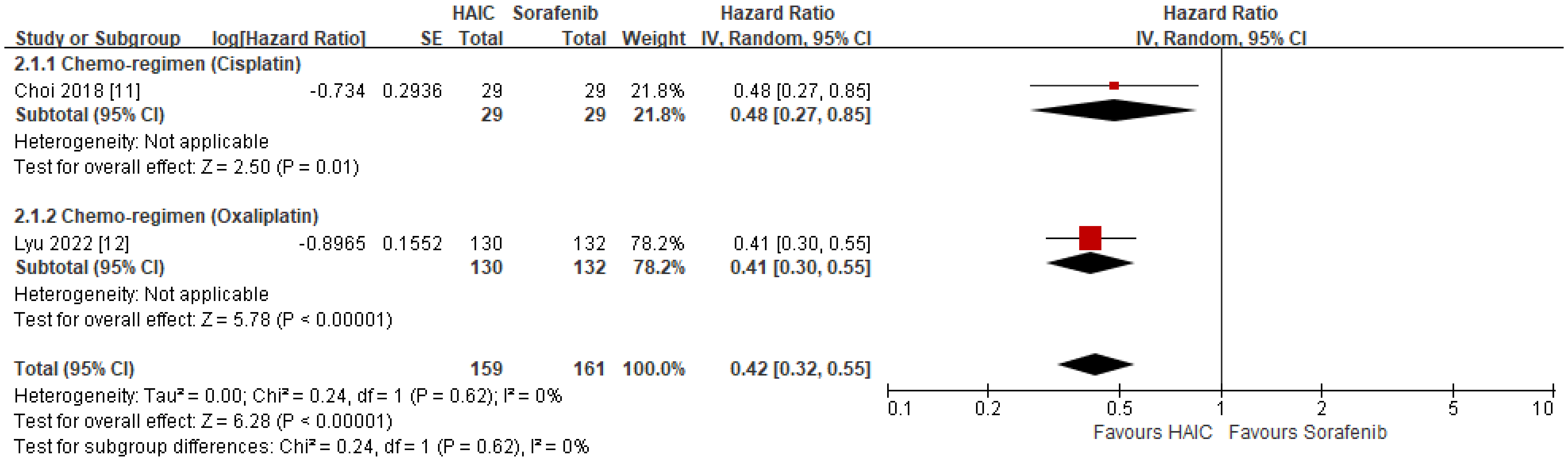

- Figure 8. HAIC vs Sorafenib; Progression-free survival

Study or Subgroup	log[Hazard Ratio]	SE	HAIC Total	Sorafenib Total	Weight	Hazard Ratio IV, Random, 95% CI
**2.2.1 Chemo-regimen (Cisplatin)**						
Choi 2018 [11]	-0.4878	0.1937	14	19	41.2%	0.61 [0.42, 0.90]
**Subtotal (95% CI)**			**14**	**19**	**41.2%**	**0.61 [0.42, 0.90]**
Heterogeneity: Not applicable						
Test for overall effect: Z = 2.52 (P = 0.01)						
**2.2.2 Chemo-regimen (Oxaliplatin)**						
Lyu 2022 [12]	-0.7963	0.1441	130	132	58.8%	0.45 [0.34, 0.60]
**Subtotal (95% CI)**			**130**	**132**	**58.8%**	**0.45 [0.34, 0.60]**
Heterogeneity: Not applicable						
Test for overall effect: Z = 5.53 (P < 0.00001)						
**Total (95% CI)**			**144**	**151**	**100.0%**	**0.51 [0.38, 0.69]**
Heterogeneity: Tau² = 0.02; Chi² = 1.63, df = 1 (P = 0.20); I² = 39%						
Test for overall effect: Z = 4.41 (P < 0.0001)						
Test for subgroup differences: Chi² = 1.63, df = 1 (P = 0.20), I² = 38.8%						

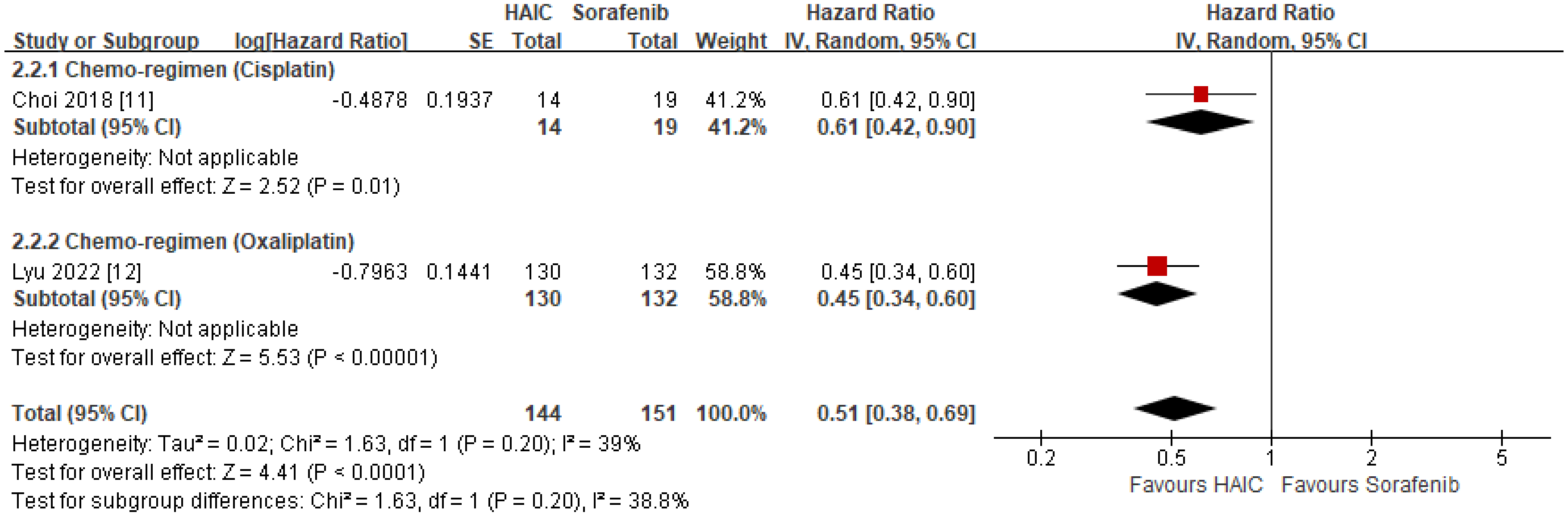

- Figure 9. HAIC vs Sorafenib; Adverse events

Study or Subgroup	HAIC Events	HAIC Total	Sorafenib Events	Sorafenib Total	Weight	Risk Ratio M-H, Random, 95% CI
**2.3.1 Chemo-regimen (Cisplatin)**						
Choi 2018 [11]	25	29	27	29	7.2%	0.93 [0.78, 1.10]
**Subtotal (95% CI)**		**29**		**29**	**7.2%**	**0.93 [0.78, 1.10]**
Total events	25		27			
Heterogeneity: Not applicable						
Test for overall effect: Z = 0.86 (P = 0.39)						
**2.3.2 Chemo-regimen (Oxaliplatin)**						
Lyu 2022 [12]	121	130	132	132	92.8%	0.93 [0.89, 0.98]
**Subtotal (95% CI)**		**130**		**132**	**92.8%**	**0.93 [0.89, 0.98]**
Total events	121		132			
Heterogeneity: Not applicable						
Test for overall effect: Z = 2.86 (P = 0.004)						
**Total (95% CI)**		**159**		**161**	**100.0%**	**0.93 [0.89, 0.98]**
Total events	146		159			
Heterogeneity: Tau² = 0.00; Chi² = 0.00, df = 1 (P = 0.95); I² = 0%						
Test for overall effect: Z = 2.99 (P = 0.003)						
Test for subgroup differences: Chi² = 0.00, df = 1 (P = 0.95), I² = 0%						

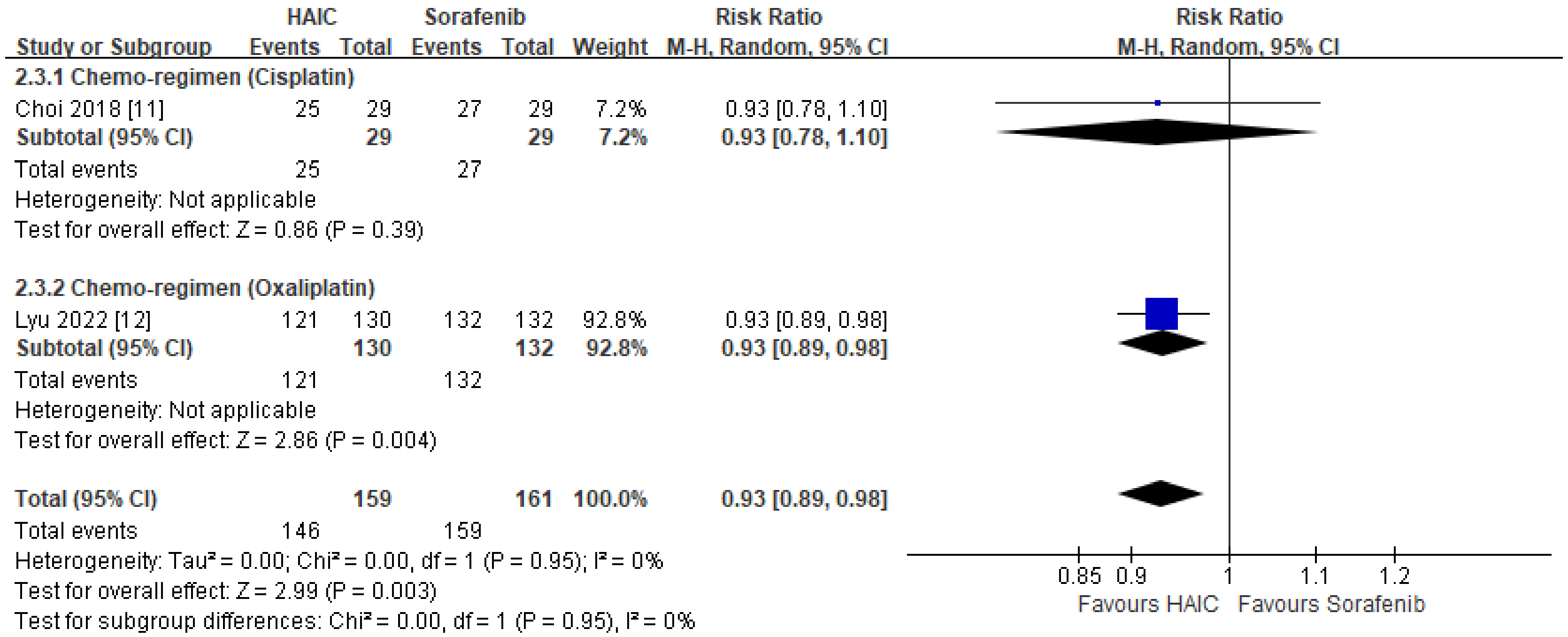

- Figure 10. HAIC vs Sorafenib; Objective response rate

	HAIC		Sorafenib			Risk Ratio
Study or Subgroup	Events	Total	Events	Total	Weight	M-H, Random, 95% CI
Choi 2018 [11]	8	29	1	29	32.5%	8.00 [1.07, 59.95]
Lyu 2022 [12]	41	130	2	132	67.5%	20.82 [5.14, 84.28]
**Total (95% CI)**		**159**		**161**	**100.0%**	**15.25 [4.84, 48.10]**
Total events	49		3			

Heterogeneity: Tau² = 0.00; Chi² = 0.60, df = 1 (P = 0.44); I² = 0%
Test for overall effect: Z = 4.65 (P < 0.00001)

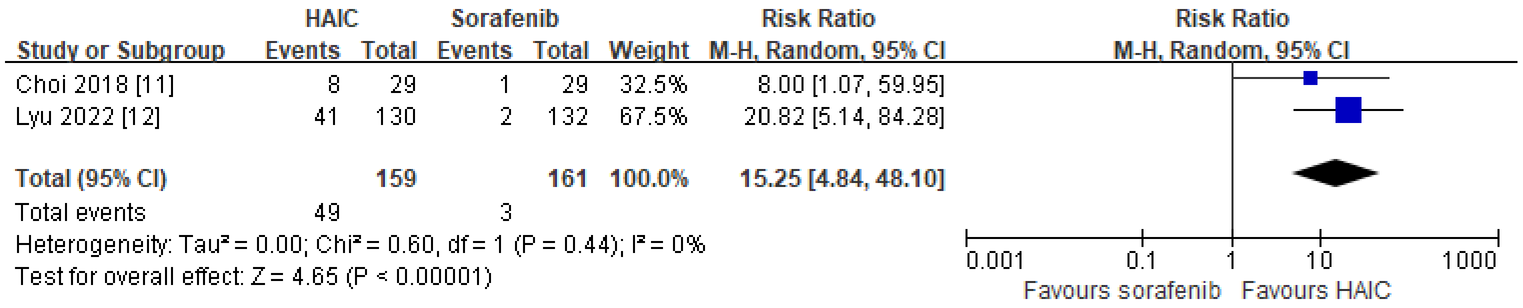

• Figure 11. HAIC vs Sorafenib; Disease control rate

	HAIC		Sorafenib			Risk Ratio
Study or Subgroup	Events	Total	Events	Total	Weight	M-H, Random, 95% CI
Choi 2018 [11]	23	29	8	29	42.7%	2.88 [1.55, 5.34]
Lyu 2022 [12]	101	130	77	132	57.3%	1.33 [1.12, 1.58]
**Total (95% CI)**		**159**		**161**	**100.0%**	**1.85 [0.86, 3.99]**
Total events	124		85			

Heterogeneity: Tau² = 0.26; Chi² = 5.87, df = 1 (P = 0.02); I² = 83%
Test for overall effect: Z = 1.57 (P = 0.12)

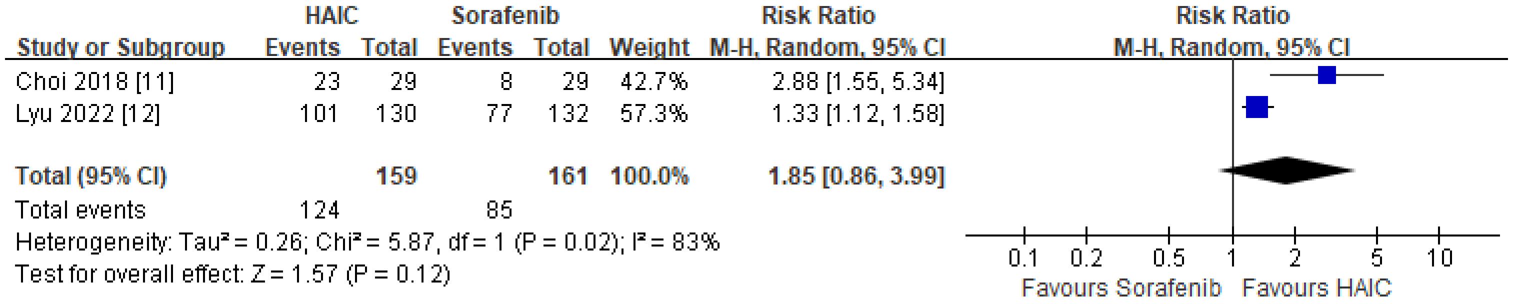

Supplement: Supplementary file 1 [file DataSheet_1.pdf]
