# Supplementary material for: Hepatic arterial infusion chemotherapy versus systemic therapy for advanced hepatocellular carcinoma: a systematic review and meta-analysis
Source: Front Oncol. 2023 Oct 10;13:1265240. doi: 10.3389/fonc.2023.1265240 (PMC10597692; doi:10.3389/fonc.2023.1265240)
Supplement: Supplementary file 2 [file DataSheet_2.pdf]

Characteristics of included studies
Ikeda 2016

**Methods**	Study design: open-labeled, randomized, active-controlled, parallel-group phase III trial<br>Study dates: November 4, 2010 - June 10, 2014<br>Setting: multicenter/Japan<br>Country: Japanese
**Participants**	Inclusion criteria:<br>(1) advanced hepatocellular carcinoma not suitable for resection, local ablation, or transarterial chemoembolization. Advanced hepatocellular carcinoma was defined as four or more tumors refractory to transarterial chemoembolization or tumors with vascular invasion or extrahepatic spread based on histological examination of biopsy samples or findings by dynamic CT, dynamic MRI, or CT scan during hepatic arteriography or arterioportography, according to American Association for the Study of Liver Diseases (AASLD) criteria<br>(2) aged 20 years or older<br>(3) life expectancy of 12 weeks or greater<br>(4) not candidates for hepatectomy, local ablation therapy, or transarterial chemoembolization<br>(5) Eastern Cooperative Oncology Group (ECOG) performance status of 0–1<br>(6) Child-Pugh score of 7 or lower (7) adequate bone marrow, liver, and renal function<br><br>Exclusion criteria:<br>(1) refractory pleural effusion or ascites<br>(2) hepatic encephalopathy<br>(3) severe and active co-morbidity or concomitant malignancy allergic reaction to iodine contrast medium precluding angiography<br>(4) pregnant and lactating females<br>(5) females of childbearing age unless using effective contraception<br>(6) unsatisfactory general condition<br><br>Estimated enrollment; 108<br><br>Age (mean): Experimental 66 (range 25-79), control 64 (range 42-78)<br><br>Gender(M/F); Experimental 56/9, control 32/9<br><br>Child-pugh class; Experimental (A/B/C) (Child 5/6/7) 38;19;8, control 27;12;2<br><br>PCT(Vp1/Vp2/Vp3/Vp4); Experimental 40 (4/9/14/13), control 17 (0/4/7/6)<br><br>Tumor clinical stage (BCLC B/C); Experimental 19 (29.2) / 46 (70.8), control 16 (39.0) / 25 (61.0)

	No of tumor(1-3/>3) ; Experimental 14 (8/5/1); 51(5/46), control 8(4/3/1); 33 (3/30)<br><br>AFP(mean, range); Experimental 223.5 (1.2-394944), control 188, (2-749412)<br><br>PIVKAII (mean, range); Experimental 1772 (10-261920), control 179, (9-1410000)<br><br>Viral (B/C)/ no-viral disease; Experimental 40 (22/18) / 25, control 29 (9/20) / 12
**Outcomes**	Primary outcomes<br>• Overall survival<br>Secondary outcomes<br>• Time to progression<br>• Adverse events<br>• Objective response rate (mRECIST for HCC)
**Intervention**	Experimental; HAIC (cisplatin) + sorafenib<br>sorafenib (Nexavar®, Bayer Health Care Pharmaceuticals; West Haven, CT, USA) was administered orally at a dose of 400 mg bid, and cisplatin (IA call®, Nippon Kayaku Co., Ltd; Tokyo, Japan) was administered concurrently at 65 mg/m2/cycle via a catheter placed in the proper, right, or left hepatic artery, or another feeding artery, every 4–6 weeks<br><br>Control; sorafenib<br>Sorafenib was administered orally at a dose of 400 mg bid
**Funding**	National Cancer Center Research and Development Fund
**Declarations of interest**	None
**Notes**	Language of publication: English

Kondo 2019

**Methods**	Study design: open-label, randomized, phase III<br>Study dates: May 2017 - May 2020<br>Setting: single center<br>Country: China
**Participants**	Inclusion criteria:<br>(1) Cytohistological confirmation is required for diagnosis of HCC<br>(2) Patients with advanced (unresectable and/or metastatic, stage C based on Barcelona-Clinic Liver Cancer [BCLC] staging classification) hepatocellular carcinoma which would not be suitable for treatment with loco-regional

therapies or have progressed following locoregional therapy such as surgical resection, percutaneous hepatic arterial embolization, radiofrequency ablation, and percutaneous interventional therapy

(3) At least one tumor lesion meeting measurable disease criteria as determined by RECIST v1.1. Lesions previously treated with local therapy, such as radiation therapy, hepatic arterial embolization, radiofrequency ablation, and percutaneous interventional therapy should not be selected unless progression is noted at baseline, in which case, these lesions would be considered as non-target lesions

(4) Current cirrhotic status of Child-Pugh class A-B, with no encephalopathy

(5) Ascites controlled by diuretics is permitted in this study

(6) Availability of a representative tumor tissue specimen (archival tumor tissue is allowed) at pre-screening

(7) Eastern Cooperative Oncology Group Scale for Assessment of Patient Performance Status ≤ 2

(8) Both men and women enrolled in this trial must use adequate barrier birth control measures during the course of the trial and 4 weeks after the completion of trial

(9) Adequate bone marrow, liver and renal function as assessed by central lab by means of the following laboratory requirements from samples within 7 days prior to procedure: Hemoglobin > 100g/L, Absolute neutrophil count >3.0 ×109/L, Neutrophil count > 1.5 ×10^9/L, Platelet count ≥ 50.0 ×10^9/L, Total bilirubin < 51 μmol/L, Alanine transaminase (ALT) and aminotransferase (AST) < 5 x upper limit of normal, Albumin > 28 g/L, Prothrombin time (PT)-international normalized ratio (INR) < 2.3, or PT < 6 seconds above control, Serum creatinine < 110 μmol/L

(10) Willing and able to comply with scheduled visits, treatment plan and laboratory tests.

Exclusion criteria:

(1) refractory pleural effusion or ascites

(2) hepatic encephalopathy

(3) severe and active co-morbidity or concomitant malignancy allergic reaction to iodine contrast medium precluding angiography

(4) pregnant and lactating females

(5) females of childbearing age unless using effective contraception

(6) unsatisfactory general condition

Screened:70; Eligible: 70

Age (mean years, SD): Experimental 72.0 (7.0), control 70.9 (9.1)

Gender (M/F); Experimental 28/7, control 27/6

Child-pugh class; Experimental (A/B/C) (Child 5/6/7) 20/11/4, control 10/19/4

	Portal v. thrombosis; Experimental 21, control 22<br><br>Tumor clinical stage<br>UICC stage III, IV; Experimental (UICC II 15/IIIA 2/IIIB 6/IIIC 2/IVA 4/IVB<br>Control (UICC II 14/IIIA 3/IIIB 7/IIIC 1 / IVA 3 / IVB 5)<br>BCLC A/B/C; Experimental (2/14/19), control (2/13/18)<br><br>AFP(mean, range); Experimental 67.3 (2-281600), control 216.7 (5-161160)<br><br>PIVKAII (mean, range); Experimental 335 (12-99800), control 1068, (14-272000)<br><br>Viral (B/C)/ no-viral disease; Experimental 24(3/21) / 11, control 24(4/20) / 10
**Intervention**	Experimental; HAIC (cisplatin) + sorafenib<br>sorafenib Nexavar® was administered orally at a dose of 400 mg bid, and cisplatin was administered concurrently at 65 mg/m^2/cycle via a catheter placed in the proper, right, or left hepatic artery, or another feeding artery, every 4–6 weeks. The sorafenib treatment in both arms was continued until tumor progression or unacceptable toxicity, and the HAIC with cisplatin was administered up to a maximum of six cycles until radiological or symptomatic tumor progression, unacceptable toxicity, or technical difficulty in repeating the HAIC.<br>Control; Sorafenib<br>sorafenib was administered orally at a dose of 400 mg bid.
**Outcomes**	Primary outcomes<br>● 1-year survival rate<br>Secondary outcomes<br>● Overall survival, 2yr survival rate, Time to progression<br>● Objective response rate, disease control rate<br>● Safety
**Funding sources**	Waksman Foundation of Japan INC
**Declarations of interest**	None
**Notes**	Language of publication: English

Kudo 2018

**Methods**	Study design: open-label, randomized, phase III<br>Study dates: May 2017 - May 2020<br>Setting: single center<br>Country: China

**Participants**	Inclusion criteria:<br>(1) advanced hepatocellular carcinoma not suitable for resection, local ablation, or transarterial chemoembolisation. Advanced hepatocellular carcinoma was defined as four or more tumours refractory to transarterial chemoembolisation or tumours with vascular invasion or extrahepatic spread based on histological examination of biopsy samples or findings by dynamic CT, dynamic MRI, or CT scan during hepatic arteriography or arterioportography, according to American Association for the Study of Liver Diseases (AASLD) criteria.<br>(2) aged 20 years or older<br>(3) life expectancy of 12 weeks or greater<br>(4) not candidates for hepatectomy, local ablation therapy, or transarterial chemoembolisation<br>(5) Eastern Cooperative Oncology Group (ECOG) performance status of 0–1<br>(6) Child-Pugh score of 7 or lower (7) adequate bone marrow, liver, and renal function<br><br>Exclusion criteria:<br>(1) another previous or current malignancy, except for curatively treated intraepithelial cervical cancer, basal cell carcinoma, superficial bladder cancer, early gastric cancer, or other early cancers with a low risk of recurrence<br>(2) had renal failure requiring haemodialysis or peritoneal dialysis<br>(3) congestive heart failure, active coronary artery disease, ischaemic heart disease, or serious cardiac arrhythmia<br>(4) poorly controlled hypertension<br>(5) active clinically serious infection (grade ≥3)<br>(6) hearing impairment<br>(7) history of HIV infection<br>(8) significant gastrointestinal bleeding within 4 weeks of study entry<br>(9) were taking a CYP3A4 inhibitor<br><br>Screened:291; Eligible: 206<br><br>Age (mean in years, SD): Experimental 66.7(10.2), control 68.1(9.1)<br><br>Gender(M/F); Experimental 89/13, control 88/15<br><br>Child-pugh class(A/B/C); Experimental 61/29/12, control 59/34/10<br><br>Tumor clinical stage; BCLC B/C; Experimental 32/710, control 27/76<br><br>AFP(mean); Experimental 440.5, control 195<br><br>PIVKAII; Experimental 2780.5, control 1487<br><br>Viral (B/C)/ no-viral disease; Experimental 73(26/47); 29, control 68(22/46);

	35
**Intervention**	Experimental; Sorafenib + HAIC (cisplatin, 5-FU)<br>Treatment was divided into 28-day cycles. All patients in both groups were treated with 400 mg sorafenib orally twice daily on days 1–28. In the hepatic arterial infusion chemotherapy combination therapy group, cisplatin was administered at a dose of 20 mg/m² per day on days 1 and 8 and fluorouracil was administered at a dose of 330 mg/m² per day on days 1–5 and 8–12 of every 28-day cycle, followed by 2 weeks off treatment.<br><br>Control; Sorafenib<br>All patients in both groups were treated with 400 mg sorafenib orally twice daily on days 1–28.
**Outcomes**	Primary outcomes<br>● OS<br>Secondary outcomes<br>● Time to progression<br>● PFS
**Funding sources**	Japanese Ministry of Health, Labour and Welfare.
**Declarations of interest**	MK has received grants from Taiho Pharmaceuticals, Chugai Pharmaceuticals, Otsuka, Takeda, Sumitomo Dainippon-Sumitomo, Daiichi Sankyo, Abbvie, Medico's Hirata, Astellas Pharma, and Bristol-Myers Squibb; grants and personal fees from MSD, Eisai, and Bayer, and is an adviser for Taiho Pharmaceuticals, Chugai Pharmaceuticals, MSD, Eisai, Bayer, Bristol-Myers Squibb, and ONO Pharmaceutical. KU, SOg, and EH have received personal fees and honoraria from Bayer. OY has received grants from Tanabe-Mitsubishi, Otsuka, Eisai, Daiichi Sankyo, MSD, Gilead, Chugai, Astellas, Takeda, and Dainippon-Sumitomo. TO has received grants from Kowa K.K. and Kyowa Hakko Kirin, grants and personal fees from Novartis, Nippon Boehringer Ingelheim, Dainippon-Sumitomo, Pfizer Jana, Inc, Bayer Yakuhin, Chugai Pharmaceuticals, Eli Lilly, Yakuruto Honsha, Ono Pharmceuticals, Eisai, AstraZeneca, Merck Serono, Baxter, Nano Carrier, Zeria Pharmaceuticals, NobelPharma, and Taiho Pharmaceuticals, and personal fees from Bristol-Myers Squibb, Nipponchemofa, EA Pharma, Fujifilm RI Pharma, Nippon Kayaku, Daiichi Sankyo, Celgene, and Teiijin Pharma. TK has received personal fees from Gilead, Bristol-Myers Squibb, MSD, and AbbVie. All other authors declare no competing interests.
**Notes**	Language of publication: English

He 2019

**Methods**	<u>Study design</u>: open-label, randomized, parallel-group phase III trial<br>Study dates: May 1, 2016 - October 10, 2017<br>Setting: multicenter/China<br>Country: China
**Participants**	Inclusion criteria:<br>(1) age 18 years or older<br>(2) biopsy confirmed hepatocellular carcinoma (not suitable for curative surgery, or local ablation)<br>(3) patients had portal vein invasion confirmed by 2 imaging techniques<br>(4) Child-Pugh A class liver function<br>(5) Eastern Cooperative Oncology Group performance status of 0 to 2<br>(6) no previous treatment for hepatocellular carcinoma<br>(7) least 1 measurable lesion according to Response Evaluation Criteria in Solid Tumors (RECIST) version 1.1,30<br>(8) adequate organfunction (white blood cellcount ≥3.0 × 10^9/L, absolute neutrophil count ≥1.5 × 10^9/L, platelet count ≥75 × 10^9/L, aspartate transaminase and alanine transaminase ≤5 × upper limit of the normal, creatinine clearance rate of ≤1.5 × upper limit of the normal, and left ventricular ejection ≥45%)<br><br>Exclusion criteria:<br>(1) hepatic decompensation, including esophageal or gastric variceal bleeding or hepatic encephalopathy<br>(2) central nervous system metastases<br>(3) a known medical history of HIV infection<br>(4) pregnancy or breastfeeding<br>(5) other invasive malignant diseases<br><br>Screened: 818; Eligible: 247<br><br>Age (mean in years, range): Experimental 49.0(41-55), control 49(40-56)<br><br>Gender (M/F); Experimental 111/14, control 112/10<br><br>Child-pugh class (A/B/C); Experimental 125;0;0, Control 122;0;0<br><br>No of Tumor; Experimental (single/multi) 30; 95, control 33; 89<br><br>AFP (mean, range); Experimental 5922 (142.6-56200.5), control 6666.5 (86.8-49609.8)<br><br>Viral (B/C)/ no-viral disease; Experimental 106 (100/6); 19, control 106(99/7);16

**Intervention**	Experimental; HAIC(cisplatin) ± sorafenib<br>Treatment was divided into 3-week cycles. Patients in both groups were treated with 400mg sorafenib orally twice daily on days 1 through 21, and HAIC regimen was performed every 3 weeks. The following regimen was administered via hepatic artery: oxaliplatin, 85 mg/m2, from hour 0 to 2 on day 1; leucovorin, 400mg/m2, from hour 2 to 3 on day 1; fluorouracil, 400mg/m2, bolus at hour 3; and 2400 mg/m2 over 46 hours on days 1 and 2. After HAIC was completed, the catheter and sheath were removed. Repetitive catheterization was performed in the next HAIC cycle.<br><br>Control ; Sorafenib<br>Treatment was divided into 3-week cycles. Patients in both groups were treated with 400mg sorafenib orally twice daily on days 1 through 21, and HAIC regimen was performed every 3 weeks.
**Outcomes**	Primary outcomes;<br>● OS<br>Secondary outcomes;<br>● PFS<br>● ORR<br>● adverse events
**Funding sources**	National Key R&D Program of China (2017YFA0505803), the National Natural Science Foundation of China (No. 81625017,No.81572385), National Science and Technology Major Project of China (2018ZX10302205), the Fundamental Research Funds for the Central Universities of China (No. 16ykjc36)
**Declarations of interest**	None
**Notes**	Language of publication: English

Zheng 2022

**Methods**	Study design: open-label, randomized, phase II trial<br>Study dates: June 2017 - November 2019<br>Setting: single center<br>Country: China
**Participants**	Inclusion criteria:<br>(1) inoperable advanced primary HCC with major PVTT, including tumor thrombosis in the main trunk (Vp4) and first branch (Vp3) of the portal vein (PVTT was graded according to the 2010 guidelines of the Japan Society of Hepatology)

	(2) histologic or clinical diagnosis of HCC<br>(3) at least one measurable lesion<br>(4) Child-Pugh class A disease<br>(5) no history of intra-arterial or systemic chemotherapy or other systemic therapies<br>(6) 18–75 years of age<br>(7) Eastern Cooperative Oncology Group performance status of 0–2<br>(8) life expectancy of 2 months or more<br>(9) adequate organ function (hemoglobin level 90 g/L; absolute neutrophil count ≥1.5×10^9/L; platelet count ≥100×10^9/L; alanine aminotransferase and aspartate aminotransferase levels 2.5-fold or less of the upper limit of normal serum total bilirubin level two fold or less of the upper limit of normal serum creatinine level 1.5-fold or less of the upper limit of normal; and serum albumin level ≥30 g/L).<br><br>Exclusion criteria:<br>(1) Have had prior chemotherapy with Oxaliplatin or Fluorouracil or Sorafenib.<br>(2) receiving any other investigational agents<br>(3) have a diagnosis of hepatic encephalopathy<br>(4) have a diagnosis of sclerosing cholangitis<br>(5) have a diagnosis of Gilbert's disease<br>(6) have clinical ascites<br>(7) have any uncontrolled concurrent illness including, but not limited to, ongoing or active infection, symptomatic congestive heart failure, unstable angina pectoris, cardiac arrhythmia, uncontrolled diabetes mellitus and hypertension, or psychiatric illness/social situations that would limit compliance with study requirements<br>(8) other malignancy except localized basal cell or squamous cell skin cancer in the past 5 years<br>(9) pregnant or lactating<br>(10) Patient Allergic to Iodine contrast medium<br>(11) Uncontrolled severe coagulation disorders (INR < 1.5 in patients not on warfarin therapy)<br><br>Screened:86; Eligible: 64<br><br>Age (mean in years, SD): Experimental 56(11), control 55(10)<br><br>Gender (M/F); Experimental 30/2, control 31/1<br><br>Child-pugh class (A/B); Experimental 28/4, control 27/5<br><br>Portal v. thrombosis (Vp3/Vp4); Experimental 32 (14/18), control

	32 (14 /18)<br><br>No of Tumor (single/multi); Experimental 15/17, Control 12/20<br><br>AFP (mean, range); Experimental 310.5 (117.5-11351), control 655.2 (78.2-74277)<br><br>Viral (B/C)/ no-viral disease; Experimental 30(28/2);2, control 32(29/3);0
**Intervention**	Experimental; sorafenib+HAIC (Oxaliplatin, 5-FU)<br>A treatment cycle lasted 28 days. All patients in both groups received oral sorafenib (Bayer) (400 mg twice daily on days 1–28). The 3cir-OFF HAIC protocol involved oxaliplatin (35 mg/m2 at hours 0–2) and 5-FU (600 mg/m2 at hours 2–24) on days 1–3 for three circulations every 4 weeks. Leucovorin (folinic acid, 200 mg/m2) was injected intravenously for 2 hours from the beginning of the 5-FU infusion on each day. The patients in the sorafenib plus HAIC group received a maximum of six cycles of HAIC and were given sorafenib thereafter. Sorafenib was administered at a 50% dosage during the 72 hours of HAIC.<br><br>Control; Sorafenib<br>A treatment cycle lasted 28 days. All patients in both groups received oral sorafenib (Bayer) (400 mg twice daily on days 1–28).)
**Outcomes**	Primary outcomes;<br>● OS<br>Secondary outcomes;<br>● ORR<br>● PFS<br>● Safety
**Funding sources**	Beijing Hospitals Authority Clinical Medicine Development of Special Funding Support (ZYLX202117), Beijing Natural Science Foundation (7212198), National Natural Science Foundation of China (82172039)
**Declarations of interest**	None
**Notes**	Language of publication: English

Choi 2018

**Methods**	Study design: Randomized, prospective, comparative study<br>Study dates: January 2013 - May 2016

	Setting: multicenter/Korea<br>Country: Korean
**Participants**	Inclusion criteria:<br>(1) age 18–70 years<br>(2) first diagnosed advanced HCC or TACE refractory advanced HCC with portal vein tumor thrombosis (Vp3 or Vp4 PVTT)<br>(3) an Eastern Cooperative Oncology Group (ECOG) performance status of 0 or 1<br>(4) Child–Turcotte–Pugh (CTP) score of 5–7<br>(5) appropriate bone marrow function such as white blood cell count ≥ 4.0 × 10^3/μL, platelet count ≥ 60 × 10^3/μL, absolute neutrophil count (ANC) ≥ 1.5 × 10^3/μL;<br>(6) serum creatinine level of ≤ 1.5 mg/dL<br>(7) no blood coagulation disorders<br>(8) no extrahepatic primary malignancy or metastasis.<br><br>Exclusion criteria:<br>(1) other concurrent serious medical condition(s) such as underlying cardiac or renal disease, infectious disease<br>(2) pregnant women, lactating women<br>(3) other chemotherapy treatments such as systemic chemotherapy<br>(4) history of gastrointestinal bleeding within 2 weeks of enrollment<br><br>Screened; 63, eligible; 58<br>Age (mean in years , SD); experimental 60.3(9.5), control 60.2(range 7.3)<br>Gender (M/F); Experimental 25/4, control 27/2<br>Child-pugh class (A/B/C) (Child 5/6/7); Experimental 27;2;0, control 25;4;0<br>PVT (Vp3/Vp4); Experimental 29 (10/19), control 29 (11/18)<br>Tumor clinical stage (UICC stage III/IV); Experimental 10/19, control 11/18<br>No of tumor (1-3/>3); Experimental 25/4, control 21/8<br>AFP (mean, SD); Experimental 260, (3.6-84604.6), control 130.8, (2.0-225971)<br>Viral (B/C)/ no-viral disease; Experimental 21(21/0); 8, control 23(18/5); 6
**Outcomes**	Primary outcomes;<br>● OS<br>● Time to progression<br>Secondary outcomes;<br>● Treatment related toxicity
**Intervention**	Experimental; HAIC (cisplatin, 5-FU)<br>The chemotherapeutic agents were cisplatin (cispuran®; Donga pharm.,

	Seoul, Korea; 60 mg/m2 for two hours on day two) and 5-fluorouracil (5-FU®; Joong-wae, Seoul, Korea; 500 mg/m2 for 5 h on days one to three). Prior to cisplatin infusion, intravenous hydration was performed to prevent nephrotoxicity. Chemotherapeutic agents were administered every 3–4 weeks through the hepatic artery, and we evaluate patient's responses every two cycles; we evaluated patients' responses after two cycles of treatment using computed tomography (CT) or magnetic resonance imaging (MRI), and if the treatment was seen as effective, we extended the observation to a maximum of six cycles.<br><br>Control ; sorafenib<br>administered as an 800 mg daily oral dose; we evaluated patients' responses after 3 months of treatment
**Note**	Language of publication: English

Lyu 2022

**Methods**	Study design: open-label, randomized, phase III<br>Study dates: May 2017 - May 2020<br>Setting: single center<br>Country: China
**Participants**	Inclusion criteria:<br>(1) Cytohistological confirmation is required for diagnosis of HCC<br>(2) Patients with advanced (unresectable and/or metastatic, stage C based on Barcelona-Clinic Liver Cancer [BCLC] staging classification) hepatocellular carcinoma which would not be suitable for treatment with loco-regional therapies or have progressed following locoregional therapy such as surgical resection, percutaneous hepatic arterial embolization, radiofrequency ablation, and percutaneous interventional therapy<br>(3) At least one tumor lesion meeting measurable disease criteria as determined by RECIST v1.1. Lesions previously treated with local therapy, such as radiation therapy, hepatic arterial embolization, radiofrequency ablation, and percutaneous interventional therapy should not be selected unless progression is noted at baseline, in which case, these lesions would be considered as non-target lesions.<br>(4) Current cirrhotic status of Child-Pugh class A-B, with no encephalopathy<br>(5) Ascites controlled by diuretics is permitted in this study

(6) Availability of a representative tumor tissue specimen (archival tumor tissue is allowed) at pre-screening

(7) Eastern Cooperative Oncology Group Scale for Assessment of Patient Performance Status ≤ 2

(8) Both men and women enrolled in this trial must use adequate barrier birth control measures during the course of the trial and 4 weeks after the completion of trial

(9) Adequate bone marrow, liver and renal function as assessed by central lab by means of the following laboratory requirements from samples within 7 days prior to procedure: Hemoglobin > 100g/L, Absolute neutrophil count >3.0 $\times 10^9$/L, Neutrophil count > 1.5 $\times 10^9$/L, Platelet count ≥ 50.0 $\times 10^9$/L, Total bilirubin < 51 µmol/L, Alanine transaminase (ALT) and aminotransferase (AST) < 5 x upper limit of normal, Albumin > 28 g/L, Prothrombin time (PT)-international normalized ratio (INR) < 2.3, or PT < 6 seconds above control, Serum creatinine < 110 µmol/L

(10) Willing and able to comply with scheduled visits, treatment plan and laboratory tests

Exclusion criteria:

(1) Received any prior systemic chemotherapy or molecular-targeted therapy for HCC such as sorafenib.

(2) Previous local therapy completed less than 4 weeks prior to the dosing and, if present any related acute toxicity > grade 1.

(3) Any contraindications for hepatic arterial infusion procedure: Impaired clotting test (platelet count < 60000/mm3, prothrombin activity < 50%). Renal failure / insufficiency requiring hemo-or peritoneal dialysis. Known severe atheromatosis. Known uncontrolled blood hypertension (> 160/100 mm/Hg).

(4) any other malignancies within the last 3 years before study start.

(5) History of HCC tumor rupture

(6) severe encephalopathy

(7) Patients with known active bleeding (e.g. from GI ulcers, esophageal varices) within 2 months prior to baseline/screening visit or with history or evidence of inherited bleeding diathesis or coagulopathy

(8) Clinically significant (CTC grade 3 or 4) venous or arterial thrombotic disease within past 6 months.

(9) History of cardiac disease: Congestive heart failure >New York Heart Association (NYHA) class 2 (refer to Appendix 13.9). Active coronary artery disease (CAD) (myocardial infarction more

	than 6 months prior to study entry is allowed). Cardiac arrhythmias (>Grade 2 NCI-CTCAE Version 4.0) which are poorly controlled with anti-arrhythmic therapy or requiring pace maker. Uncontrolled blood hypertension (> 160/100 mm/Hg).<br>(10) Serious, non-healing wound, ulcer, or bone fracture.<br>(11) History of abdominal fistula, GI perforation, or intra-abdominal abscess within past 6 months prior to study treatment.<br>(12) Clinically significant third space fluid accumulation (i.e., ascites requiring tapping despite use of diuretic or pleural effusion that either required tapping or is associated with shortness of breath).<br>(13) Patients who have undergone major surgical procedure, open biopsy, or significant traumatic injury within 4 weeks of the start of protocol treatment.<br>(14) History of a bone marrow or solid organ transplant.<br>(15) Use of biologic response modifiers, such as G-colony stimulating factor (CSF), within 3 weeks prior to start of study drug. (G-CSF and other hematopoietic growth factors may be used in the management of acute toxicity such as febrile neutropenia when clinically indicated or at the discretion of the investigator; however, they may not be substituted for a required dose reduction).<br>(16) Subjects taking chronic erythropoietin are permitted provided no dose adjustment is undertaken within 1 month prior to the study or during the study.<br>(17) Any other condition that would, in the Investigator's judgment, contraindicate patient's participation in the clinical study due to safety concerns or compliance with clinical study procedures, e.g., infection/inflammation, intestinal obstruction, unable or unwilling to swallow medication, social/ psychological issues, etc.<br>(18) Unable to undergo either contrast-enhanced magnetic resonance imaging (MRI) or contrast-enhanced computed tomography (CT).<br>(19) Known history of human immunodeficiency virus (HIV) seropositivity. HIV testing is not required as part of this study.<br>(20) Patients who have received any other investigational agents within a period of time that is less than the cycle length used for that treatment or equal to 4 weeks (whichever is shorter) prior to starting study drug and recovered from any side effects to grade 1 or less.<br>(21) Pregnant or nursing (lactating) women, where pregnancy is

defined as the state of a female after conception and until the termination of gestation, confirmed by a positive human chorionic gonadotropin (hCG) laboratory test.

(22) Women of child-bearing potential, defined as all women physiologically capable of becoming pregnant, unless they are using highly effective methods of contraception during dosing and for 7 days after permanently discontinuing HAIF and/or sorafenib treatment.

(23) Highly effective contraception methods include: Total abstinence (when this is in line with the preferred and usual lifestyle of the patient. Periodic abstinence (e.g., calendar, ovulation, symptothermal, post-ovulation methods) and withdrawal are not acceptable methods of contraception. Female sterilization (have had surgical bilateral oophorectomy with or without hysterectomy) or tubal ligation at least six weeks before taking study treatment. In case of oophorectomy alone, only when the reproductive status of the woman has been confirmed by follow up hormone level assessment. Male sterilization (at least 6 months prior to screening). For female patients on the study the vasectomized male partner should be the sole partner for that patient.

Combination of any two of the following (a+b or a+c, or b+c):

Use of oral, injected or implanted hormonal methods of contraception or other forms of hormonal contraception that have comparable efficacy (failure rate <1%), for example hormone vaginal ring or transdermal hormone contraception. Placement of an intrauterine device or intrauterine system. Barrier methods of contraception: Condom or Occlusive cap (diaphragm or cervical/vault caps) with spermicidal foam/gel/film/cream/vaginal suppository. In case of use of oral contraception women should have been stable on the same pill for a minimum of 3 months before taking study treatment. Women are considered post-menopausal and not of child bearing potential if they have had 12 months of natural (spontaneous) amenorrhea with an appropriate clinical profile (e.g. age appropriate, history of vasomotor symptoms) or have had surgical bilateral oophorectomy (with or without hysterectomy) or tubal ligation at least six weeks ago. In the case of oophorectomy alone, only when the reproductive status of the woman has been confirmed by follow up hormone level assessment is she considered not of child bearing potential. Sexually active males unless they use a condom during intercourse while receiving treatment and for 7

	days after stopping study treatment and should not father a child in this period. A condom is required to be used also by vasectomized men in order to prevent delivery of the drug via seminal fluid. Subjects unable to suffer the discomfort of the HAI procedure (e.g. pain, claustrophobia, noise). Any contraindication for sorafenib, oxaliplatin, leucovorin, or fluorouracil administration. Any agents which could affect the absorption or pharmacokinetics of the study drugs. Known or suspected allergy to the investigational agents or any agent given in association with this study.<br><br>Screened:551; Eligible: 262<br><br>Age (mean in years, SD): Experimental 54 (45-61), control 53(45-62)<br><br>Gender(M/F); Experimental 115/15, control 123/9<br><br>Child-pugh class (A/B); Experimental 88/42, control 93/39<br><br>PCT(Vp1-3/ Vp4) ; Experimental 52/37, control 55/28<br><br>Tumor clinical stage BCLC (B/C); Experimental 5/125, control 9/123<br><br>No of tumor(1-3/ >3) ; Experimental (43/87), Control (55/77)<br><br>AFP(mean, SD); Experimental 337.8(28.5-12902.75), Control 304.2(15.3-3086.5)<br><br>Viral (B/C)/ no-viral disease; Experimental 122(120/2);8, control 118(114/4);14
**Intervention**	Experimental; HAIC-FO<br>After the biopsy, each patient received an artery catheter procedure guided by digital subtraction angiography.<br>Then, the FOLFOX (oxaliplatin 130 mg/m2, leucovorin 200 mg/m2, fluorouracil 400 mg/m2, and fluorouracil 2,400 mg/m2) regimen was sequentially infused through the catheter every cycle (3 weeks).<br><br>Control ; Sorafenib<br>standard prescription of 400 mg sorafenib orally twice daily
**Outcomes**	Primary outcomes;<br>● OS<br>Secondary outcomes;<br>● PFS<br>● intrahepatic tumor PFS

	• ORR<br>• DCR
**Funding sources**	Supported by the National Natural Science Foundation of China (No.81901850, Ning Lyu; No. 82072022 and No. 81771956, Ming Zhao)
**Declarations of interest**	None
**Notes**	Language of publication: English

Characteristics of excluded studies

Kawaoka 2016

**Reason for exclusion**	Wrong study design (retrospective study)

jRCTs061210065

**Reason for exclusion**	Ongoing study, no result

NCT05198609

**Reason for exclusion**	Ongoing study, no result

Tzoracoleftherakis 1999

**Reason for exclusion**	Wrong measured outcomes(inappropriate tumor response criteria, staging), lack of patient information

ChiCtr 2021

**Reason for exclusion**	Ongoing study; no result, wrong comparator (TACE combined)

JPRN-UMIN000008710 2012

**Reason for exclusion**	Ongoing study, no result

Kamimura 2017

**Reason for exclusion**	Wrong comparator (TACE combined)

NCT04191889

**Reason for exclusion**	Ongoing study, no result

Wu 2015

**Reason for exclusion**	Wrong comparator (HAIC/interferon vs palliative care)

JPRN-jRCTs031180019 2018

**Reason for exclusion**	Wrong study design (single arm study)

Excluded study reference

1. Kawaoka T, Aikata H, Hatooka M, Honda F, Nakano N, Nakamura Y, Kobayashi T, Fukuhara T, Hiramatsu A, Chayama K. Comparison of HAIC and sorafenib in HCC patients refractory to TACE. Hepatology International 2016;10(1):S670

2. Study of HAIC added on chemotherapy in patients with unresectable advanced Hepatocellular Carcinoma; https://trialsearch.who.int/Trial2.aspx?TrialID=JPRN-jRCTs061210065

3. Camrelizumab, Apatinib Plus HAIC Versus Camrelizumab and Apatinib for HCC With Portal Vein Invasion: a Randomized Trial; https://clinicaltrials.gov/ct2/show/NCT05198609

4. Tzoracoleftherakis EE, Spiliotis JD, Kyriakopoulou T, Kakkos SK. Intra-arterial versus systemic chemotherapy for non-operable hepatocellular carcinoma. Hepatogastroenterology. 1999;46(26):1122-5

5. Randomized phase II study comparing pegylated interferon alpha-2b combined with intra-arterial 5-fluorouracil and sorafenib alone for advanced hepatocellular carcinoma; https://rctportal.niph.go.jp/en/detail?trial_id=UMIN000008710

6. Kamimura K, Suda T, Yokoo T, Kamimura H, Kanefuji T, Tsuchiya A, Takamura M, Kawai H, Waguri N, Yamagiwa S, Terai S. Transhepatic arterial infusion chemotherapy using a combination of miriplatin and CDDP powder versus miriplatin alone in the treatment of hepatocellular carcinoma: a randomized controlled trial. BMC Cancer. 2017;17(1):322. doi: 10.1186/s12885-017-3320-7

7. A Trial of Hepatic Arterial Infusion Combined With Apatinib and Camrelizumab Versus Apatinib and Camrelizumab for C-staged Hepatocellular Carcinoma in BCLC Classification; https://clinicaltrials.gov/ct2/show/NCT04191889

8. Wu J, Huang WJ, Wang HY, Wang YF, Peng BG, Zhou Q. Arterial infusion of 5-fluorouracil combined with subcutaneous injection of pegylated interferon alpha-2b in treating unresectable hepatocellular carcinoma with portal vein tumor thrombus. Med Oncol. 2015 (3):65. doi: 10.1007/s12032-015-0491-4.

9. Ikeda M, Yamashita T, Ogasawara S, Kudo M, Inaba Y, Morimoto M, Tsuchiya K, Shimizu S, Kojima Y, Hiraoka A, Nouso K, Aikata H, Numata K, Sato T, Okusaka T, Furuse J. Phase II study of lenvatinib plus hepatic intra-arterial infusion chemotherapy with cisplatin for advanced hepatocellular carcinoma - LEOPARD trial 2018;():
https://doi.org/10.1016/j.annonc.2021.08.157

Search strategies

Ovid MEDLINE(R) and Epub Ahead of Print, In-Process & Other Non-Indexed Citations via Ovid (Daily 1946 to 26 December 2022)

1 exp Carcinoma, Hepatocellular/
2 ((Liver or hepatocell*) adj3 Carcinoma*).tw.
3 Hepatoma*.tw.
4 HCC.tw.
5 1 or 2 or 3 or 4
6 exp Infusions, Intra-Arterial/ and exp Hepatic Artery/
7 hepatic arterial infusion chemotherap*.tw.
8 HAIC.tw.
9 6 or 7 or 8
10 5 and 9
11 randomized controlled trial.pt.
12 controlled clinical trial.pt.
13 randomized.ab.
14 placebo.ab.
15 drug therapy.fs.
16 randomly.ab.
17 trial.ab.
18 groups.ab.
19 11 or 12 or 13 or 14 or 15 or 16 or 17 or 18
20 exp animals/ not humans.sh.
21 19 not 20
22 10 and 21

Embase Classic+Embase via Ovid (1947 to 26 December 2022)

#1 'liver cell carcinoma'/exp
#2 ((liver OR hepatocell*) NEAR/3 carcinoma*):ti,ab
#3 hepatoma*:ti,ab
#4 hcc:ti,ab
#5 #1 OR #2 OR #3 OR #4
#6 'intraarterial drug administration'/exp AND 'hepatic artery'/exp
#7 'hepatic arterial infusion chemotherap*':ti,ab
#8 haic:ti,ab
#9 #6 OR #7 OR #8
#10 #5 AND #9
#11 (random*:ti,ab,tt OR 'randomization'/de OR 'intermethod comparison'/de OR placebo:ti,ab,tt OR compare:ti,tt OR compared:ti,tt OR comparison:ti,tt OR ((evaluated:ab OR evaluate:ab OR evaluating:ab OR assessed:ab OR assess:ab) AND (compare:ab OR compared:ab OR comparing:ab OR comparison:ab)) OR ((open NEXT/1 label):ti,ab,tt) OR (((double OR single OR doubly OR singly) NEXT/1 (blind OR blinded OR blindly)):ti,ab,tt) OR 'double blind procedure'/de OR ((parallel NEXT/1 group*):ti,ab,tt) OR crossover:ti,ab,tt OR 'cross over':ti,ab,tt OR (((assign* OR match OR matched OR allocation) NEAR/6 (alternate OR group OR groups OR intervention OR interventions OR patient OR patients OR subject OR subjects OR participant OR participants)):ti,ab,tt) OR assigned:ti,ab,tt OR allocated:ti,ab,tt

OR ((controlled NEAR/8 (study OR design OR trial)):ti,ab,tt) OR volunteer:ti,ab,tt OR volunteers:ti,ab,tt OR 'human experiment'/de OR trial:ti,tt) NOT ('randomized controlled trial'/de OR 'controlled clinical trial'/de) NOT (((random* NEXT/1 sampl* NEAR/8 ('cross section*' OR questionnaire* OR survey OR surveys OR database OR databases)):ti,ab,tt) NOT ('comparative study'/de OR 'controlled study'/de OR 'randomised controlled':ti,ab,tt OR 'randomized controlled':ti,ab,tt OR 'randomly assigned':ti,ab,tt) OR ('cross-sectional study'/de NOT ('randomized controlled trial'/de OR 'controlled clinical study'/de OR 'controlled study'/de OR 'randomised controlled':ti,ab,tt OR 'randomized controlled':ti,ab,tt OR 'control group':ti,ab,tt OR 'control groups':ti,ab,tt)) OR ('case control*':ti,ab,tt AND random*:ti,ab,tt NOT ('randomised controlled':ti,ab,tt OR 'randomized controlled':ti,ab,tt)) OR ('systematic review':ti,tt NOT (trial:ti,tt OR study:ti,tt)) OR (nonrandom*:ti,ab,tt NOT random*:ti,ab,tt) OR 'random field*':ti,ab,tt OR (('random cluster' NEAR/4 sampl*):ti,ab,tt) OR (review:ab AND review:it NOT trial:ti,tt) OR ('we searched':ab AND (review:ti,tt OR review:it)) OR 'update review':ab OR ((databases NEAR/5 searched):ab) OR ((rat:ti,tt OR rats:ti,tt OR mouse:ti,tt OR mice:ti,tt OR swine:ti,tt OR porcine:ti,tt OR murine:ti,tt OR sheep:ti,tt OR lambs:ti,tt OR pigs:ti,tt OR piglets:ti,tt OR rabbit:ti,tt OR rabbits:ti,tt OR cat:ti,tt OR cats:ti,tt OR dog:ti,tt OR dogs:ti,tt OR cattle:ti,tt OR bovine:ti,tt OR monkey:ti,tt OR monkeys:ti,tt OR trout:ti,tt OR marmoset*:ti,tt) AND 'animal experiment'/de) OR ('animal experiment'/de NOT ('human experiment'/de OR 'human'/de)))
#12 #10 AND #11

Cochrane Library via Wiley
#1 '[mh "Carcinoma, Hepatocellular"]
#2 ((Liver or hepatocell*) near/3 Carcinoma*):ti,ab,kw
#3 'Hepatoma*:ti,ab,kw
#4 HCC:ti,ab,kw
#5 #1 or #2 or #3 or #4
#6 [mh "Infusions, Intra-Arterial"] and [mh "Hepatic Artery"]
#7 hepatic arterial infusion chemotherap*:ti,ab,kw
#8 HAIC:ti,ab,kw
#9 #6 or #7 or #8
#10 #5 and #9

Web of Science
#1 TS=(((Liver or hepatocell*) NEAR/3 Carcinoma*) OR Hepatoma* OR HCC)
#2 TS=("hepatic arterial infusion chemotherap*" OR HAIC)
#3 TS=(randomised OR randomized OR randomisation OR randomisation OR placebo* OR (random* AND (allocat* OR assign*)) OR (blind* AND (single OR double OR treble OR triple)))
#4 #1 AND #2 AND #3

ClinicalTrials.gov
Condition: Hepatocellular Carcinoma* OR Hepatoma* OR HCC
Intervention: hepatic arterial infusion chemotherap* OR HAIC

WHO ICTRP
1 (Hepatocellular Carcinoma* OR Hepatoma* OR HCC) AND (hepatic arterial infusion

chemotherap* OR HAIC)

KOREAMED
1 "Carcinoma, Hepatocellular"[MH] OR Hepatoma[TIAB] OR Hepatomas[TIAB] OR HCC[TIAB] OR ((Liver[TIAB] OR hepatocellular[TIAB]) AND (Carcinoma[TIAB] OR Carcinomas[TIAB]))

2 ("Infusions, Intra-Arterial"[MH] AND "Hepatic Artery"[MH]) OR "hepatic arterial infusion chemotherapy"[TIAB] OR HAIC[TIAB]

3 1 AND 2

KMBASE
1 ((([ALL=Hepatocellular Carcinoma*] OR [ALL=Hepatoma*]) OR [ALL=HCC]) OR [ALL=간세포암])
2 ([ALL=hepatic arterial infusion chemotherap*] OR [ALL=HAIC])
3 1 AND 2
